# A Beta Strain-Based Spike Glycoprotein Vaccine Candidate Induces Broad Neutralization and Protection against SARS-CoV-2 Variants of Concern

**DOI:** 10.1128/spectrum.02687-22

**Published:** 2023-02-27

**Authors:** Lei Cao, Jinyuan Guo, Hai Li, Hu Ren, Kang Xiao, Yan Zhang, Shuangli Zhu, Yang Song, Weijia Zhao, Dan Wu, Zhihui Chen, Yanan Zhang, Baicheng Xia, Tianjiao Ji, Dongmei Yan, Dongyan Wang, Qian Yang, Yangzi Zhou, Xiaolei Li, Zhanjun Hou, Wenbo Xu

**Affiliations:** a NHC Key Laboratory of Medical Virology and Viral Diseases, National Institute for Viral Disease Control and Prevention, Chinese Center for Disease Control and Prevention, Beijing, China; b Hwellso Biotechnology (Beijing) Co., Ltd., Beijing, China; c Dongzhimen Hospital Affiliated to Beijing University of Chinese Medicine, Beijing, China; d Center for Biosafety Mega-Science, Chinese Academy of Sciences, Wuhan, Hubei, China; University of Georgia

**Keywords:** SARS-CoV-2, spike glycoprotein, broad neutralization, variants of concern, variant-based vaccine

## Abstract

The coronavirus disease 2019 (COVID-19) pandemic is still ongoing. Severe acute respiratory syndrome coronavirus 2 (SARS-CoV-2) variants of concern (VOCs) are circulating worldwide, making it resistant to existing vaccines and antiviral drugs. Therefore, the evaluation of variant-based expanded spectrum vaccines to optimize the immune response and provide broad protectiveness is very important. In this study, we expressed spike trimer protein (S-TM) based on the Beta variant in a GMP-grade workshop using CHO cells. Mice were immunized twice with S-TM protein combined with aluminum hydroxide (Al) and CpG Oligonucleotides (CpG) adjuvant to evaluate its safety and efficacy. BALB/c immunized with S-TM + Al + CpG induced high neutralizing antibody titers against the Wuhan-Hu-1 strain (wild-type, WT), the Beta and Delta variants, and even the Omicron variant. In addition, compared with the S-TM + Al group, the S-TM + Al + CpG group effectively induced a stronger Th1-biased cell immune response in mice. Furthermore, after the second immunization, H11-K18 hACE2 mice were well protected from challenge with the SARS-CoV-2 Beta strain, with a 100% survival rate. The virus load and pathological lesions in the lungs were significantly reduced, and no virus was detected in mouse brain tissue. Our vaccine candidate is practical and effective for current SARS-CoV-2 VOCs, which will support its further clinical development for potential sequential immune and primary immunization.

**IMPORTANCE** Continuous emergence of adaptive mutations of severe acute respiratory syndrome coronavirus 2 (SARS-CoV-2) continues to challenge the use and development of existing vaccines and drugs. The value of variant-based vaccines that are capable of inducing a higher and broader protection immune response against SARS-CoV-2 variants is currently being evaluated. This article shows that a recombinant prefusion spike protein based on a Beta variant was highly immunogenic and could induced a stronger Th1-biased cell immune response in mice and was effectively protective against challenge with the SARS-CoV-2 Beta variant. Importantly, this Beta-based SARS-CoV-2 vaccine could also offer a robust humoral immune response with effectively broad neutralization ability against the wild type and different variants of concern (VOCs): the Beta, Delta, and Omicron BA.1 variants. To date, the vaccine described here has been produced in a pilot scale (200L), and the development, filling process, and toxicological safety evaluation have also been completed, which provides a timely response to the emerging SARS-CoV-2 variants and vaccine development.

## INTRODUCTION

The emergence of severe acute respiratory syndrome coronavirus 2 (SARS-CoV-2) has dramatically changed social and economic development. Mutation of certain amino acid sites on SARS-CoV-2 has enhanced the virus’s transmissibility, pathogenicity, and immune evasion ability and correspondingly reduced the effectiveness of vaccines, therapeutic drugs, and diagnostic tools. Currently, these variants are classified as variants of concern (VOCs) and variants of interest (VOIs) (1). Five strains of SARS-CoV-2 were designated VOCs (Alpha, Beta, Gamma, Delta, and Omicron).

Some VOCs not only have decreased susceptibility to antivirals and monoclonal antibodies but also have reduced neutralizing capacity with sera from convalescent patients and vaccinated individuals. The neutralizing antibody titers against live viruses of the Alpha, Beta, and Delta strains were 2 to 3, 3 to 6, and 17 to 30 times lower than those of the Wuhan-Hu-1 strain (wild-type [WT]), respectively (2). Neutralizing antibodies against the Omicron strain were minimal or undetectable when induced by mRNA vaccines, adenovirus vector vaccines, and inactivated vaccines (3–5). Sera from convalescing patients cannot neutralize the Omicron variant either. Many key amino acid mutations in the S protein (D614G, N501Y, E484K, and K417N/T, etc.), especially in the receptor-binding domain (RBD) and furin cleavage site, may result in various degrees of immune escape. Therefore, there is an urgent need to develop variant-based expanded spectrum vaccines against pandemic variants, including Omicron, to prevent COVID-19.

Some studies have suggested that SARS-CoV-2 T-cell immunity acquired by S-focused COVID-19 vaccines or previous infection would retain broadly robust cellular immunity against different VOCs (6, 7). T-cell immunity plays an important role in controlling SARS-CoV-2 infection and reducing disease severity. In SARS-CoV-2 patients, the S glycoprotein is a good target for both CD4^+^ and CD8^+^ T-cell responses, which demonstrates the importance of cellular immunity for the current development of SARS-CoV-2 vaccines (8). According to World Health Organization data, 35% of the vaccine candidates in clinical trials are based on a protein subunit platform, accounting for the highest proportion among different vaccine strategies. However, subunit vaccines generally have poor immunogenicity and require adjuvants to enhance their ability to generate an immune response. CpG Oligonucleotides (CpG) is a mature adjuvant with wide application in herpes simplex virus types 1 and 2, as well as cancer vaccines. As a TLR9 receptor agonist, CpG can increase the number of CD8^+^ T cells by inducing the maturation of dendritic cells and inducing the Th1-cellular immune response at the same time. However, CpG easily diffuses away from the injection site, which leads to a low effective concentration. Luckily, CpG is well adsorbed to aluminum hydroxide (Al), which avoids CpG diffusion from the injection site. This combination enhances antigen uptake, activation by dendritic cells, and induced inflammatory responses by Al at the injection site, but there is a reduction in eosinophil production relative to that induced by Al alone (9). Studies have shown that the combination of Al and pattern recognition receptor agonists stimulates better immune responses than using Al alone (10).

Data from our research show that a recombinant prefusion spike protein based on a Beta strain was highly immunogenic and could induce protective antibodies (neutralizing antibodies against WT and different VOCs: the Beta, Delta, and Omicron variants) and cell-mediated responses that were protective against challenge with the SARS-CoV-2 Beta variant.

## RESULTS

### Characterization of S-TM.

**FIG 1:**
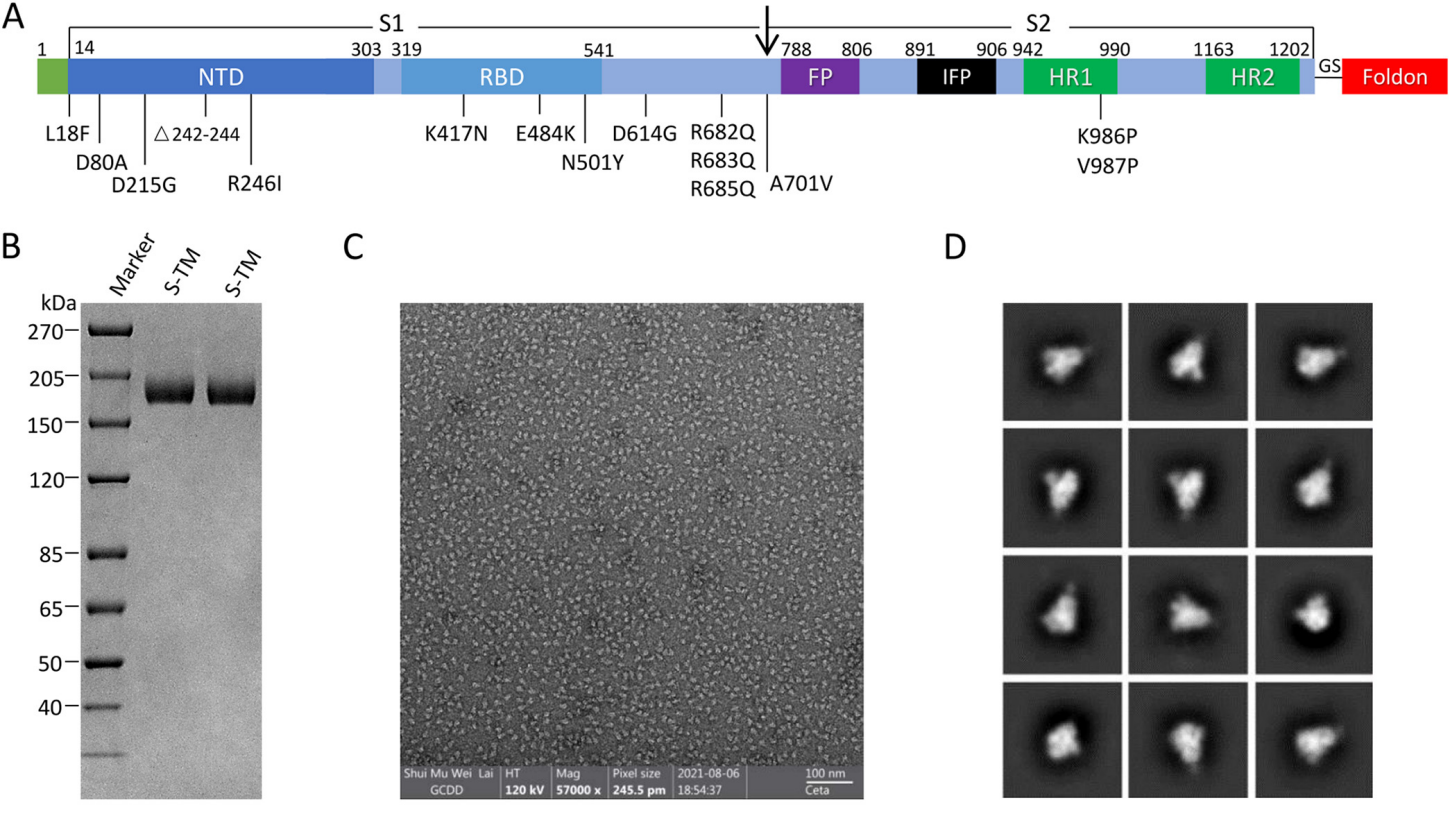
Spike trimer protein (S-TM) design, expression, and characterization. (A) Linear diagram of the SARS-CoV-2 spike (S) protein ectodomain based on the protein sequence of Beta variant. S1 subunit has the N-terminal domain (NTD, dark blue) and receptor-binding domain (RBD, blue); S2 subunit has the fusion peptide (FP, purple), internal fusion peptide (IFP, black), heptad repeat 1 and heptad repeat 2 (HR1 and HR2, green), a flexible protein linker (GS), and a foldon sequence (red). The protein sequence mutations based on the Beta strain different from the WT strain were generated as follows: L18F, D80A, D215G, R246I, K417N, E484K, N501Y, D614G, A701V, R682Q, R683Q, R685Q, K986P, and V987P, with deletion of 242 to 244 amino acids. (B) SDS-PAGE analysis shows purified uncleaved S-TM protein expressed by CHO-K1 cells, with a molecular weight of around 180 kDa. (C) Negative-stain electron microscopy (EM) images of ectodomain of S-TM. (D) Two-dimensional images (performed by cisTEM) of S-TM show clear triangular protein particles.

The S-TM was based on the WT SARS-CoV-2 S-gene encoding an extracellular region of 1,211 amino acids. Fourteen mutations were generated based on the Beta VOC strain. Only a few mutations were generated at the furin cleavage site (682-RRAR-685 to 682-QQAQ-685) and K986P and V987P for structure stabilization. A T4 foldon was inserted at the C terminus of S-TM for self-trimerization (Fig. 1A). S-TM has a molecular weight of approximately 180 kDa according to SDS-PAGE analysis after expression and purification (Fig. 1B). By transmission electron microscopy (TEM) and two-dimensional class averaging, the ectodomain of S-TM, formulated in PB buffer (20 mM Phosphate Buffer, 150 mM NaCl, and 0.02% polysorbate 80 (PS80)), pH 6, showed a prefusion trimeric conformation with good uniformity and dispersion with a 19.6-nm length and a 13.23-nm width (Fig. 1C and D). The stability of antigen stands for a critical factor that contributes to the viability of a vaccine candidate. The electron microscope photos showed that the purified S-TM still presented a homogeneous trimeric structure with good dispersion when stored at 4°C (for 87 days) and −80°C (for 112 days), respectively (Fig. S1A to D).

### S-TM + Al + CpG induced high binding antibodies and cross-neutralizing antibodies against SARS-CoV-2 VOCs.

**FIG 2:**
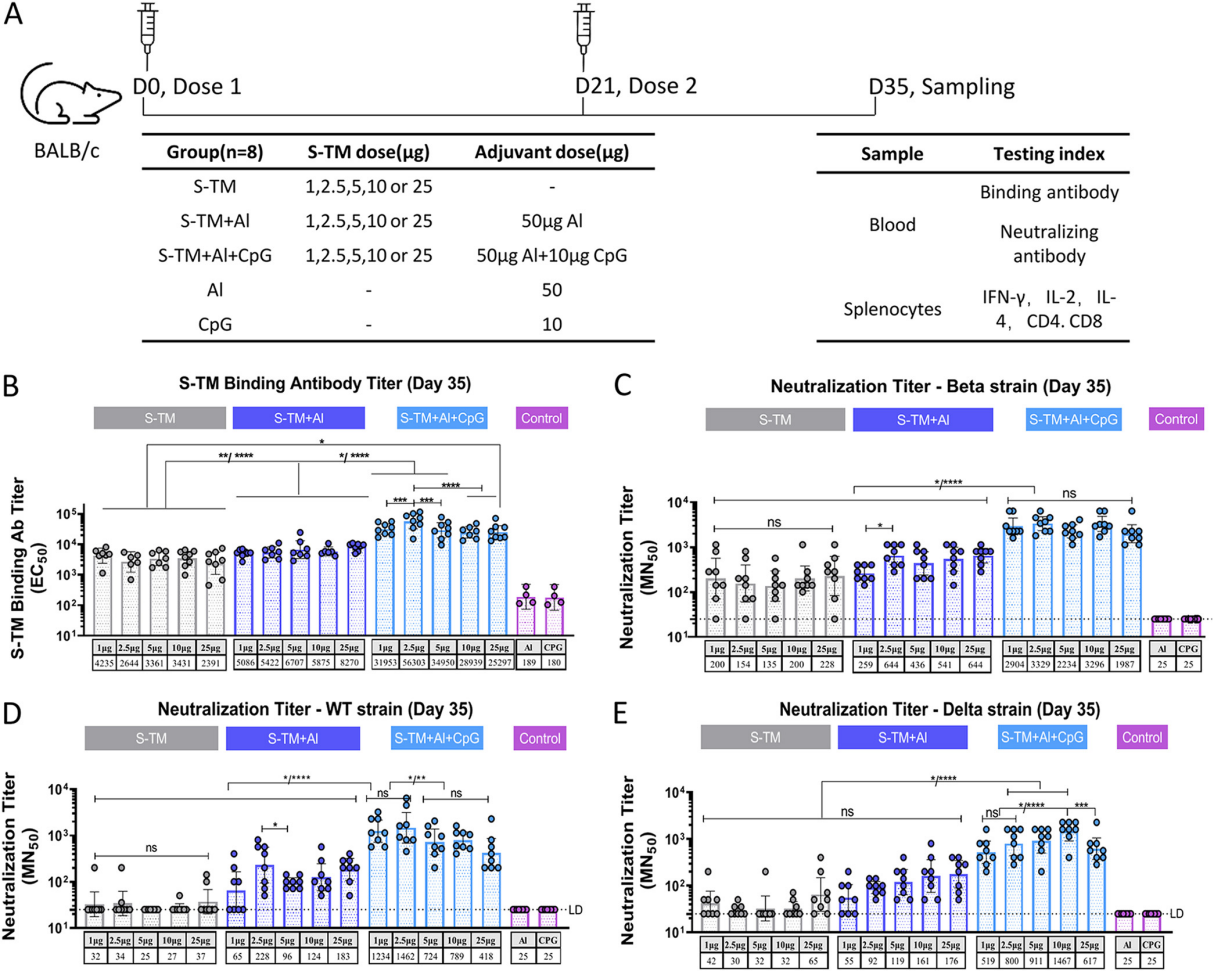
Antibody induced upon immunization with S-TM based on the Beta variant. (A) Schematic representation of the study schedule. BALB/c mice (*n* = 8) were immunized with 1, 2.5, 5, 10, and 25 μg S-TM that was nonadjuvanted or adjuvanted with 50 μg Al or 50 μg aluminum hydroxide (Al) + 10 μg CpG twice on days 0 and 21 with primary analysis for humoral- and cell-mediated immunogenicity conducted on day 35 for blood and splenocyte samples. (B) S-TM binding antibody titers with ELISA. (C to E) Neutralizing antibody titers against Beta (C), wild-type (WT) (D), and Delta (E) strains with live SARS-CoV-2 assay. The points represent individual mice. For binding antibody (Ab), the bars represent the geometric mean with 95% CI of the antibody titer (EC_50_), and comparisons were made using one-way analysis of variance (ANOVA) with Tukey’s multiple-comparison test. For the neutralizing antibody, the limitation of detection (LD) was initial dilution fold (1:25), data lower than the LD were considered initial dilution and were used as fold limited detection for calculation and plotting. Bars represent the geometric mean with 95% CI of the antibody titer, and the comparisons were made using ordinary one-way ANOVA multiple-comparison test. *P* values < 0.05 were considered significant. S-TM, spike trimer protein; CpG, CpG Oligonucleotides; MN50, the neutralization titer for 50% neutralization of viral infection; IFN, interferon; IL, interleukin; ns, not significant.

Mice were immunized intramuscularly (IM) with various doses of S-TM (1, 2.5, 5, 10, and 25 μg/dose) on days 0 and 21 with S-TM, S-TM + Al, or S-TM + Al + CpG. Blood samples were collected 14 days after the second immunization for S-TM binding and neutralizing antibody tests (Fig. 2A). S-TM binding antibody titers in the S-TM + Al + CpG groups were significantly higher than those in the S-TM and S-TM + Al groups at the corresponding antigen dose levels (except for the 10 or 25 μg S-TM + Al + CpG group) (Fig. 2B). This phenomenon also appeared in neutralization results, which in the S-TM + Al + CpG groups were the highest at the corresponding antigen dose levels (Fig. 2C to E), especially for the Beta strain, and significant differences were observed in all antigen-dose groups (geometric mean titer [GMT]: S-TM + Al + CpG 1,987 to 3,329, S-TM + Al 259 to 644, S-TM 135 to 228, *P* < 0.05) (Fig. 2C). Overall, the mice vaccinated with S-TM + Al + CpG groups had significantly higher humoral immunogenicity than that of nonadjuvanted and Al-adjuvanted groups. These results indicate the effect of CpG on humoral immunogenicity.

**FIG 3:**
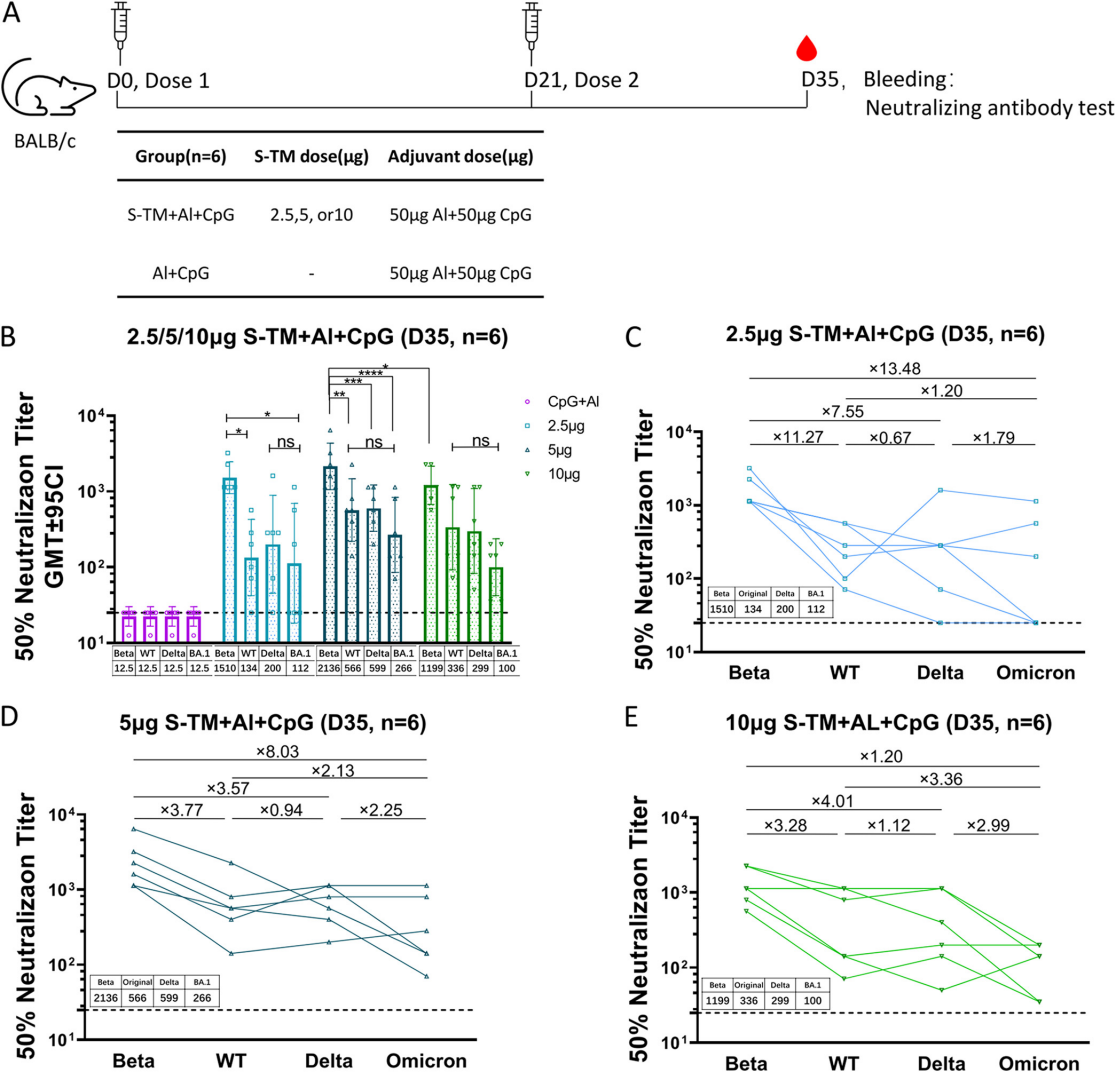
Cross-neutralizing based on live severe acute respiratory syndrome coronavirus 2 (SARS-CoV-2) neutralization titer after two doses of immunization with S-TM based on the Beta variant. (A) Schematic representation of the study schedule. BALB/c mice (*n* = 6) were immunized with 2.5, 5, and 10 μg S-TM that was adjuvanted with 50 μg Al + 50 μg CpG or only adjuvant without antigen twice on days 0 and 21; blood was collected at 14 days after the second vaccination for determination of neutralizing antibody titers. (B) Neutralizing antibody titer against the Beta, WT, Delta, and Omicron strains induced by S-TM + Al + CpG. (C to E) Neutralizing antibody titer changes among four virus strains in 2.5 μg (C), 5 μg (D), and 10 μg (E) groups. Points represent individual mice. Bars represent the geometric mean with 95% CI of the antibody titer, and comparisons were made using two-way ANOVA with Tukey’s multiple-comparison test. *P* values < 0.05 were considered significant. S-TM, spike trimer protein; Al, aluminum hydroxide; CpG, CpG Oligonucleotides; ns, not significant. GMT, geometric mean titer.

To further analyze the spectrum of serum-neutralizing antibodies against VOCs, we compared the cross-neutralization ability in the S-TM + Al + CpG group toward WT and VOCs together. As shown in Fig. S2A, the GMTs of serum-neutralizing antibodies against the Beta strain was higher than against the Delta and WT strains (*P* < 0.05). Neutralizing antibody levels were similar for WT and Delta (*P >* 0.05). Immunization with the Beta-based S-TM + Al + CpG vaccine showed the highest neutralizing antibody against the parent strain, while reduced antibody against Delta VOC (Fig. S2A to F). Encouragingly, the serum of mice that received 5- or 10-μg doses maintained quite a high level of neutralizing antibodies against Delta, with degrees of neutralization that declined less than 3-fold compared to that against Beta (Fig. S2D and E). According to the above-described experimental results, we immunized mice with 2.5, 5, and 10 μg S-TM + Al + 50 μg CpG (one-fifth of the dose of the finished preparation) and continued to explore the neutralizing antibody change between Omicron and other strains. Another group of BALB/c mice (15 to 19 g, female) was randomly grouped (*n* = 6/group) and immunized intramuscularly on days 0 and 21 with S-TM + Al + CpG (Fig. 3A). Neutralizing antibody titers against the Beta strain (GMT 1,199 to 2,136) were significantly higher than those against the Delta strain (GMT 200 to 599) and the Omicron strain (GMT 100 to 266) at the corresponding antigen doses (Fig. 3B). Furthermore, neutralizing antibody titers against the Delta strain and the Omicron strain dropped 5.04-fold (3.57 to 7.55) and 11.17-fold (8.03 to 13.48), respectively, compared to that of the Beta strain. Neutralizing antibody titers against the Omicron strain dropped 2.35-fold (1.79 to 2.99) compared to that of the Delta strain (Fig. 3C to E). Overall, in all groups, the 5 μg S-TM + Al + CpG group showed the highest neutralizing antibody titers against the four strains, and they were significantly higher than those of the 10 μg S-TM + Al + CpG group (*P* < 0.05). The 5 μg S-TM + Al + CpG group showed the least neutralizing antibody titer drop against the Omicron strain compared to the Beta strain (8.03) (Fig. 3C).

### S-TM + Al + CpG induced a Th1-biased cell-mediated immune response.

**FIG 4:**
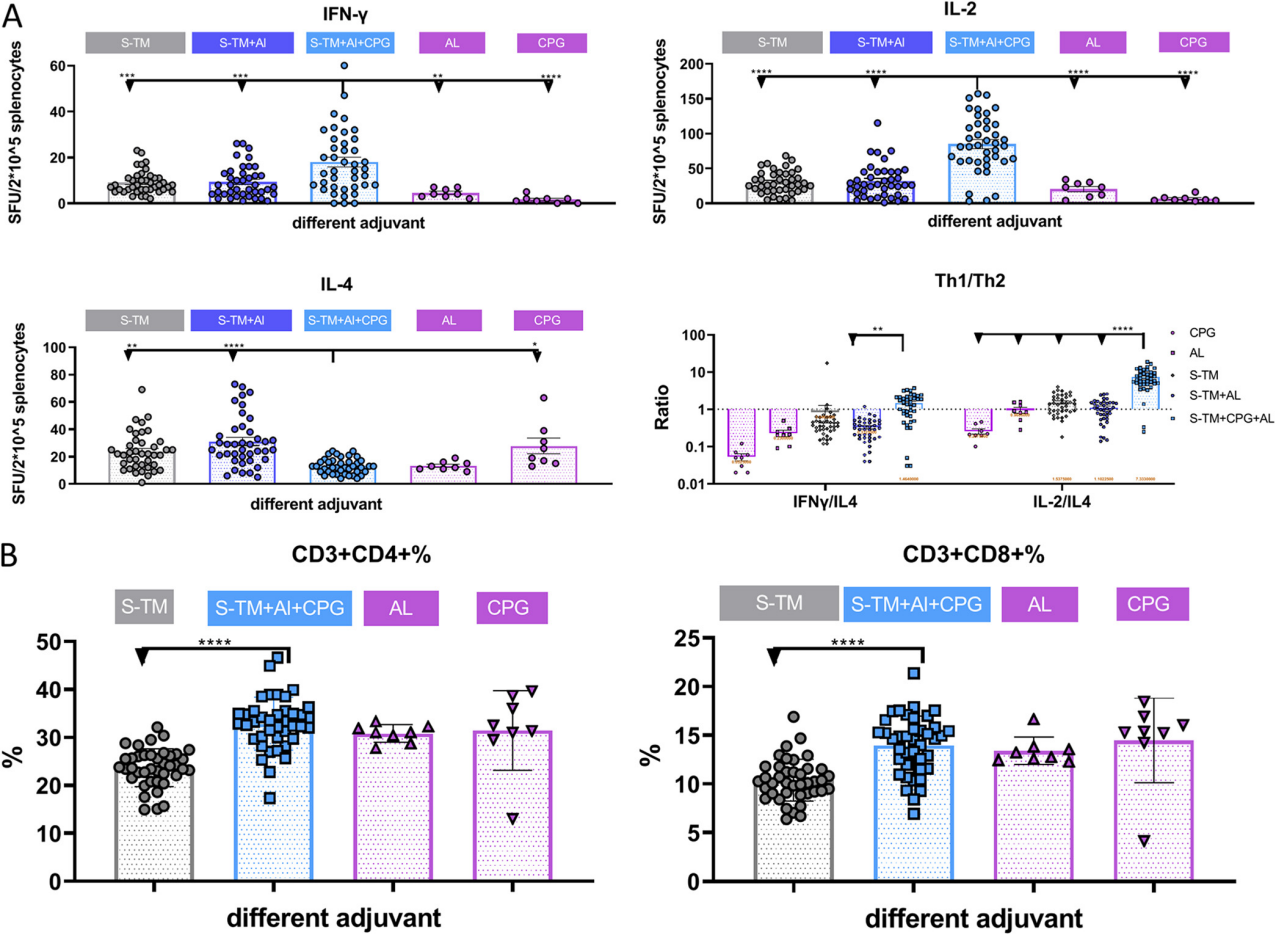
Cell-mediated immunity in different adjuvant group upon immunization with S-TM based on the Beta variant. BALB/c mice (*n* = 8) were immunized with 1, 2.5, 5, 10, and 25 μg S-TM that was nonadjuvanted or adjuvanted with 50 μg Al or 50 μg Al + 10 μg CpG twice on days 0 and 21 (different dose group was shown in Fig. S3). (A) The amount of immune cells in spleen secreting IL-2, IFN-γ, and IL-4 cytokines was determined, and the ratios of IFN-γ/IL-4 and IL-2/IL-4 were calculated 14 days after the second vaccination. (B) Frequency of CD3^+^CD4^+^ and CD3^+^CD8^+^ T cells in different groups. Bars indicate means ± standard error of the mean (SEM). Comparisons were made using one-way ANOVA with Tukey’s multiple-comparison test. *P* values < 0.05 were considered significant. S-TM, spike trimer protein; Al, aluminum hydroxide; CpG, CpG Oligonucleotides; SFU, spot-forming units; IL, interleukin; IFN, interferon.

After two IM immunizations with various doses of S-TM (1, 2.5, 5, 10, and 25 μg/dose) on days 0 and 21 with S-TM, S-TM + Al, or S-TM + Al + CpG, splenocytes were harvested at 14 days, followed by stimulation with spike peptide pool detection of Th1 (interleukin 2 [IL-2] and interferon γ [IFN-γ]) and Th2 (IL-4) for the study of antigen-specific cell-mediated immunity (Fig. 2A). The S-TM + Al + CpG group induced more IL-2- and IFN-γ-secreting cells than the Al-adjuvanted and CpG-adjuvanted groups, which indicates that S-TM + Al + CpG induced spike (S)-specific IL-2- and IFN-γ-secreting cells, leading to antigen-specific cell-mediated immunity (Fig. 4A). We calculated the ratio between them to evaluate the type of cell-mediated immune response. The ratio of Th1 to Th2 cytokine-secreting cells from the same mouse (IFN-γ/IL-4 and IL-2/IL-4) was higher in the S-TM + Al + CpG group than in the S-TM + Al group (1.5 versus 0.3 and 7.3 versus 1.1; *P* < 0.002 and *P* < 0.00001) (Fig. 4A). These results show that CpG can balance the Th2-biased cell-mediated immune response and induce a Th1-biased cell-mediated immune response. In addition, only IFN-γ showed an antigen dose-dependent change in the S-TM + Al + CpG group, and dose saturation was observed at 25 μg S-TM in IL-2- and IL-4-secreting cells (Fig. S3A).

For CD4^+^ and CD8^+^ T-cell-mediated immunity, the S-TM + Al + CpG group induced a higher ratio of mature CD4^+^ and CD8^+^ T cells than the S-TM group (Fig. 4B). The ratio of CD8^+^ but not CD4^+^ T cells showed an antigen dose-dependent change in the S-TM + Al + CpG group, and a saturated dose was observed at 25 μg S-TM (Fig. S3B).

### S-TM + Al + CpG protects H11-K18-hACE-2 mice from beta strain infection.

**FIG 5:**
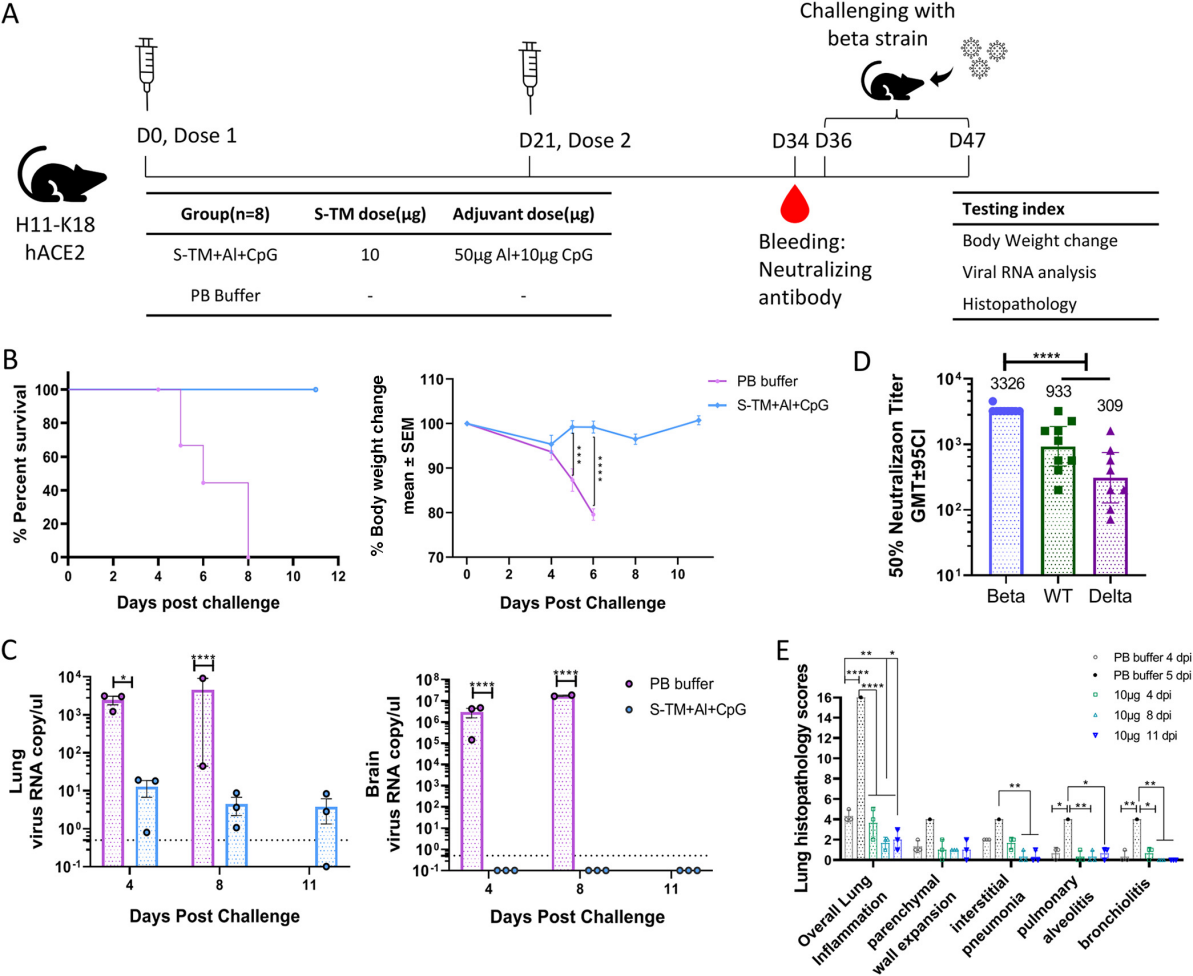
Protection against SARS-CoV-2 Beta strain infection in H11-K18-hACE-2 mice after immunization with S-TM based on Beta variant. (A) Schematic representation of the challenge schedule. H11-K18-hACE-2 mice (*n* = 9) were immunized with S-TM + Al + CpG (10 μg) with or without adjuvants (only PB buffer) twice on days 0 and 21, and serum samples were taken at day 34 for immunogenicity assays. Two weeks after the second immunization, the mice were challenged with the SARS-CoV-2 Beta strain. Body weight was tracked, and the animals were euthanized for tissue sampling at 4 to 11 days postinfection (dpi). (B) Survival rates and changes in body weight after virus challenge in H11-K18-hACE-2 mice. (C) RNA copies in the lung and brain were detected by reverse transcription (RT)-PCR. The detection threshold (0.5 copies/μL) is shown by the dotted line in each panel. RNA copies below the threshold were set to 1/5 threshold for calculation and plotting. (D) Neutralizing antibody titer against the Beta, WT, and Delta strains 13 days after the second immunization. (E) Lung pathology scoring in mice at 4, 5, 8m or 11 dpi. For RNA copies and lung pathology scores, the bars indicate mean ± SEM, and statistical significance was calculated with two-way ANOVA with Tukey’s multiple-comparison test. For statistical analysis of antibody titers, the results are presented as the geometric mean with 95% confidence interval and statistical significance calculated with one-way ANOVA with Tukey’s multiple-comparison test. *P* values < 0.05 were considered significant. S-TM, spike trimer protein; Al, aluminum hydroxide; CpG, CpG Oligonucleotides; PB buffer: 20 mM Phosphate Buffer, 150 mM NaCl, and 0.02% polysorbate 80 (PS80), pH 6.

H11-K18-hACE-2 mice were vaccinated with 10 μg S-TM + Al + CpG or PB buffer to evaluate the induction of protective immunity against challenge with the SARS-CoV-2 Beta strain (Fig. 5A). In the PB buffer group, mice showed trembling, poor mobility and balance, weight loss, and death at 4 days postinfection (dpi); most of the mice experienced 20% weight loss at 6 dpi; and 100% of the mice were dead at 8 dpi (Fig. 5B). However, all mice in the S-TM + Al + CpG group maintained their body weight and survived until 11 dpi (Fig. 5B). In terms of virus load, PB buffer-treated mice had 10^3^ copies/μL in the lungs and 10^6^ to 10^7^ copies/μL in the brain at 4 and 8 dpi. The mice immunized with S-TM + Al + CpG had almost undetectable virus copies in the brain and decreased 2 to 3 log in the lung at 4 and 8 dpi (Fig. 5C). Notably, 1 of 3 immunized mice at 4, 8, and 11 dpi almost had no virus detected in their lung tissue. Thus, S-TM + Al + CpG effectively stimulated H11-K18-hACE-2 mice at 13 days after the second vaccination and induced humoral immunogenicity, with high neutralizing antibody titers against the Beta strain (GMT 3,326), WT strain (GMT 933), and Delta strain (GMT 309) (Fig. 5D).

**FIG 6:**
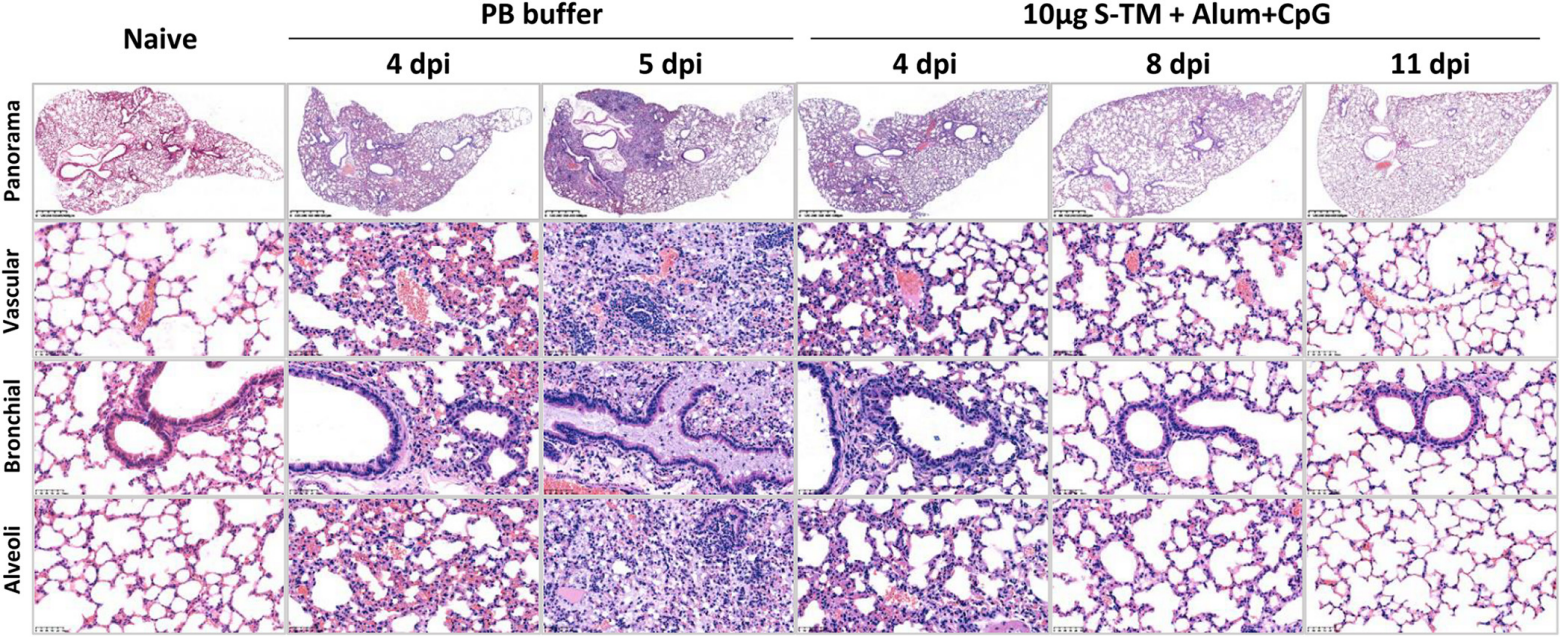
Histopathological analysis of SARS-CoV-2 Beta strain infection with or without S-TM-immunized H11-K18-hACE2 mice. The mice (*n* = 9) were immunized with S-TM + Al + CpG (10 μg) with or without adjuvants (only PB buffer) with two doses spaced 14 days apart. Two weeks after the second immunization, the mice were challenged with the SARS-CoV-2 Beta strain. Lungs were collected 4, 5, 8, or 11 dpi. The representative images of histopathology in lungs from the vaccinated group and the nonvaccinated control group are displayed. S-TM, spike trimer protein; Al, aluminum hydroxide; CpG, CpG Oligonucleotides; dpi, days postinfection; PB buffer: 20 mM Phosphate Buffer, 150 mM NaCl, and 0.02% polysorbate 80 (PS80), pH 6.

For pulmonary histology analysis, due to the death of mice in the PB buffer group after infection, the samples were collected for the analyses at 4 and 5 dpi. In the S-TM + Al + CpG group, the samples were collected at 4, 8, and 11 dpi. As seen from the pathological sections and scores, the lung pathology of mice infected by the Beta strain was mainly manifested by thickening of the alveolar wall and diffused alveolar damage interstitial pneumonia, accompanied by mild bronchitis and alveolitis (Fig. 5E). At 4 dpi, PB buffer-treated mice showed thickening of the alveolar septa and interstitial inflammation with hemangiectasis, hyperemia, interstitial edema, and infiltration of lymphocytes (Fig. 6). At 5 dpi, the thickening of the alveolar septa and interstitial inflammation were aggravated, and there was more infiltration of lymphocytes and monocytes (Fig. 6). The S-TM + Al + CpG-immunized mice showed a significant reduction in pathological reactions and lung inflammation at 4 to 11 dpi (Fig. 6). These results indicate that S-TM + Al + CpG immunization can delay disease progression with only mild lung inflammation during Beta strain infection and may relieve pathological symptoms in the following days.

## DISCUSSION

In this study, the S-TM protein expressed in CHO cells with a foldon (GYIPEAPRDGQAYVRKDGEWVLLSTFL) existed in the form of a stable and uniform trimer. The foldon, located at the C terminus of phage T4 fibrin, contains 27 amino acids (containing a large number of hydrophobic amino acids) and forms a trimer based on intermolecular hydrogen bonds and hydrophobic forces (11). This method has also been applied to research on the structure of the S protein in the early stages of COVID-19 (12). Consistent with the earlier observations, we also found that in low pH conditions, S-TM revealed more homogenous and stable trimer than at pH 7 to 8. There is a pH switch domain (residues 617 to 639) that undergoes dramatic conformational changes at different pH values and that may account for the enhanced stability of the spike trimer at lower pH (13). The biological mechanism of S-TM structural arrangement in different pH values still needs to be further studied. To date, our vaccine has been produced in a pilot scale (200L), and the development and filling processes have also been completed. The antigen produced on the pilot scale has good dispersibility, stability, and homogeneity.

We showed that two doses of S-TM + Al + CpG were effective in eliciting neutralizing activity against various SARS-CoV-2 strains, including WT, Beta, Delta, and Omicron. Although there was no specific antigen dose-dependent relationship in the immunization of BALB/c mice with S-TM +Al + CpG, a 25-μg antigen dose did not show increased titer of both binding and neutralizing antibodies, indicating that 25 μg had reached saturation. In addition, 1 μg of S-TM + Al + CpG induced an even higher neutralizing antibody titer than S-TM alone or S-TM + Al at the 10- or 25-μg antigen doses, which indicates the potential for a more than 10-fold or greater dose-sparing effect provided by the Al + CpG adjuvant. This is consistent with other reports that CpG can enhance vaccine effectiveness through strong B-cell activation, resulting in enhanced antibody production (14).

Although our Beta variant-based vaccine candidate showed the highest neutralizing antibodies against the homologous strains, it was observed that mice produced neutralizing antibodies against Delta that were almost equal to or slightly higher than the WT strain and neutralizing antibodies against Omicron that were only 1.2 to 3.36 times lower than the WT strain. Studies have revealed that the current vaccines (adenovirus vectors, mRNA, and subunit vaccines) targeting Beta mutated SARs-CoV-2 RBD, or spike proteins elicited higher levels of binding and cross-neutralizing antibodies against variants than against the WT SARS-CoV-2 (15–18). While vaccines based on the WT strain produced neutralizing antibodies against the Beta and Delta strains that were approximately 7 to 29 times lower than that of the WT strain, for Omicron cross-neutralizing antibodies, it was approximately 26 times lower or even provided no detectable titer against the Omicron variant (19). Therefore, we speculate that our S-TM based on the Beta strain has better immunogenicity and can stimulate mice to produce a broader cross-neutralizing antibody response to VOC strains (Beta, Delta, and Omicron strains) than the vaccines based on the WT strain. One study reported that the levels of neutralization antibodies induced by an Omicron-specific booster dose were lower, indicating that an Omicron-specific vaccine may not provide sufficient immunity or protection (20). The results of *in vivo* experiments show that those mutations decreased the antigenicity of Omicron in general (21, 22). This raises the possibility and significance of exploring Beta-specific vaccines as an effective vaccine against Omicron. Our S-TM candidate induces strong broad neutralizing antibodies, potentially due to some shared mutation sites (K417, E484, N501, and D614G) in its sequence with Omicron. Some studies have shown that the cross-neutralizing antibodies of Beta and Omicron variants mainly depend on the 417N/T and 501Y mutations (23). In addition to the RBD regions, other conserved regions in spike proteins may provide broadly neutralizing antibodies (24).

Adjuvants can effectively enhance and regulate the immune response intensity and bias of vaccines. Aluminum adjuvants have been used in many vaccines to induce bias toward the Th2 immune response. Enhanced respiratory disease (ERD) induces a Th2-biased immune response induced by the FI-RSV vaccine combined with aluminum adjuvant in children. CpG is an oligodeoxynucleotide containing a nonmethylated cytosine guanine dinucleotide that binds to TLR9 receptors in cells to induce an immune response that is Th1 oriented. Synergistic use with aluminum adjuvant can induce the body to produce a balanced immune response, and a CpG library can be formed *in vivo* through the action of aluminum adjuvant, thus prolonging its action time *in vivo*. As expected from the known Th1 bias in the response to CpG-containing adjuvants, BALB/c mice that received S-TM + Al + CpG induced more IL-2- and IFN-γ-secreting cells than those who received S-TM + Al, and the same magnitude of immune responses was observed in another preclinical subunit protein vaccine based on the Beta strain (25, 26). In addition, S-TM + Al + CpG induced a Th-1-biased cell-mediated immune response and inhibited an Al-directed Th-2-biased cell-mediated immune response, which reduced the possibility of a Th-2-biased cell-mediated immune response associated with vaccine-associated enhanced respiratory disease (VAERD), indicating the safety of our vaccines (27, 28).

Furthermore, S-TM + Al + CpG induced more CD4^+^ and CD8^+^ T cells than S-TM in BALB/c mice, which is associated with better virus clearance and an improved clinical outcome (29–32). Although Omicron has many more mutations than the other VOC strains, a study showed that 80% of conserved CD4^+^ and CD8^+^ T-cell epitopes were observed on vaccinees from different vaccine suppliers (33). In our study, we used the SARS-CoV-2 S peptide based on the WT strain to stimulate splenocytes, and we still observed a significant increase in S antigen-specific IL-2-, IFN-γ-, and IL-4-secreting cells, which reconfirmed the conservativeness of T-cell epitopes against SARS-CoV-2 (34–37). This gives us a more optimistic cross-reaction against other VOCs, especially for the Omicron strain.

In our study, we demonstrated that our vaccine candidate S-TM + Al + CpG protects against lethal infection of H11-k18 mice, a severe disease model (38, 39). For the PB control group, inoculation of K18-hACE2 mice with a 50% tissue culture infective dose (TCID_50_)/mouse SARS-CoV-2 of 25 (based on doses leading to 100% mouse death in our preliminary study) resulted in 100% mortality, lung disease with signs of interstitial pneumonia, immune infiltration, and extensive spread of the virus to the central nervous system and the brain (Fig. 5 and 6; Fig. S4). Although the challenge dose was lower than that used in a K18 mouse study using a 100 TCID_50_/mouse challenge dose, the survival days, weight changes, and viral load after challenge were not significantly different from those used in our study (Fig. 5; Fig. S4), suggesting minimal impact of the challenge dose on mouse model establishment (40). This finding indicates that a SARS-CoV-2 mouse model was successfully established in our study, which sets the foundation for subsequent virus challenge. Although SARS-COV-2 primarily targets the respiratory system, some patients with COVID-19 also develop extrarespiratory symptoms, including neurological manifestations. SARS-CoV-2 can infect neurons in human brain organoids (41). In our study, immunization with S-TM + Al + CpG not only significantly inhibited viral replication and reduced pathological lesions in the lung but also prevented severe symptoms and death in mice by almost completely blocking viral replication in the brain (Fig. 5; Fig. S4), which precluded potential antibody-dependent enhancement (ADE) effect, and we can further speculate that our vaccine candidate is effective not only in preventing SARS-CoV-2 infection but also in preventing virus entry into the central nervous system and is highly effective in preventing severe illness and death.

H11-k18 mice immunized with S-TM + Al + CpG produced a high titer of neutralizing antibodies against Beta SARS-CoV-2, which played an important role in resistance to virus infection. In our study, BALB/c mice immunized with S-TM + Al + CpG produced cross-neutralizing antibodies against the VOC Omicron BA.1 strain. Therefore, it can be inferred that our vaccine candidate is also protective against Omicron strain infection. All H11-K18 mice immunized twice with the finished dosage form of S-TM + Al + CpG survived challenge with the Beta, Delta, and Omicron BA.1 strains, while all the mice in the control group died. As Omicron BA.2 and BA.5 variants emerged, we further explored the protective effect against Omicron BA.2.12.1 and BA.5 variant nonlethal challenge with other hACE2-KI mice. Within expected, the mice immunized twice with the finished dosage form of S-TM + Al + CpG showed significant reduction in lung nucleic acid load and pulmonary pathology (unpublished data). In mice, the S-TM + Al + CpG elicits protection against SARS-CoV-2 variant infection with no evidence of ADE.

At the time of this article's composition, most of the population has received a COVID-19 vaccine as a primary series based on the WT strain (42, 43). However, reduction or loss of detectible levels of neutralizing antibody against the variants at ~6 months might be indicative of waning protection and likely due to further affinity maturation of B cells and alteration of the available antibody repertoire (44). The value of variant-based vaccines that can induce a higher and broader immune response against SARS-CoV-2 at booster vaccination was being evaluated. A clinical trial showed that a heterologous boosting with Beta-based MVB.1.351 vaccine resulted in a higher neutralizing antibody titer against the Beta, Delta, and Omicron BA.1 variants than did the Wuhan-Hu-1 strain-based vaccine formulation (45); this demonstrated the high value of variant-adapted vaccines capable of inducing a higher and broader immune response against SARS-CoV-2 at booster vaccination. The long-term antibody level could be a critical factor that contributes to the application prospect of the present vaccine candidate. Our vaccine candidate is presumed to have great potential on providing broad and long-term protection as booster vaccination, which could be ideal for alleviating the burden of the global COVID-19 pandemic and is very promising for further development from the bench to the clinic.

## MATERIALS AND METHODS

### Animal studies, facilities, and ethics statements.

Specific pathogen-free (SPF) BALB/c female mice (15 to 19 g) and H11-K18-hACE-2 female mice (5 weeks) were purchased from Beijing HFK Bioscience Co., Ltd., and Gempharmatech Co., Ltd., and were kept under standard pathogen-free conditions at Yikang (Beijing) Pharmaceutical Technology Co., Ltd., and the animal experimental center in the BSL-3 at the Chinese National Institute for Viral Disease Control and Prevention, China CDC, respectively. All mouse experiments were approved by the Animal Care Welfare Committee of Yikang (Beijing) Pharmaceutical Technology Co., Ltd. (Animal Experimental Ethical Inspection Form YK-2021-005), National Institute for Viral Disease Control and Prevention (IVDC), China CDC (Animal Experimental Ethical Inspection Form 20210830068 and BSL-3 Animal Experimental Ethical Inspection Form 121051018) and were conducted according to the Guide for Care and Use of Laboratory Animals.

### Virus strains, cells, and expression vectors.

The WT SARS-CoV-2 strain (Hub01), SARS-CoV-2 Beta/B.1.351 strain (GD2021), and SARS-CoV-2 Delta/B.1.617.2 strain (CQ2021) were isolated and stocked in IVDC, China CDC. The SARS-CoV-2 Omicron/BA.1 strain (Omi-2) was courtesy of the University of Hong Kong. Vero cells were stocked in IVDC, China CDC, and were cultured in minimum essential medium (MEM) (GIBCO) supplemented with 2% (vol/vol) fetal bovine serum (FBS) (GIBCO) and 1% (vol/vol) penicillin/streptomycin (HyClone). Viral stocks were prepared in Vero cells, and the titer of the stock was determined by TCID_50_ assays using Vero cells. CHO-K1 cells were provided by Beijing JOINN Biologics Co. Ltd. The spike protein expression vector (ZY-5.0) was constructed by Beijing JOINN Biologics Co. Ltd., and it was transfected into CHO-K1 cells, which were screened for stable cell lines expressing the spike protein. Virus amplification, titer determination of stock, mouse challenge study, and tissue homogenization were performed in the BSL-3 of IVDC, China CDC.

### Protein design, expression, and structure.

To generate the recombinant spike construct based on the Beta variant (S-TM), the following point mutations were introduced: L18F, D80A, D215G, R246I, K417N, E484K, N501Y, D614G, A701V, and deletion of 242–244. To maintain the stability of the prefusion conformation, R682Q, R683Q, R685Q, K986P, and V987P were also introduced. For the trimer conformation, the foldon sequence, GYIPEAPRDGQAYVRKDGEWVLLSTFL, derived from the native T4 phage fibritin to stabilize a trimeric hairpin propeller and a flexible protein linker, GS, was substituted at the C-terminal domain. Full-length S genes were cloned between the EcoRI-HindIII sites in the ZY-5.0 vector, named ZYB0029. The expression vector ZYB0029 was transfected into CHO-K1 cells using a GenPulser (Bio-Rad, USA). With increasing concentrations of MSX, stable clones were screened by monoclonal sorting. After harvesting the clarified cell culture medium, we purified recombinant S-TM by consecutive chromatographic steps (cation exchange (POROS XS, Thermo), hydrophobic chromatography (Phenyl HP), compound anion chromatography (Mix-A), and cation exchange (SP Mustang)). The S-TM was formulated at PB buffer (20 mM Phosphate Buffer, 150 mM NaCl, and 0.02% polysorbate 80 (PS80), pH 6). The expression and purification of S-TM were performed in the good laboratory practice (GLP) laboratory at Beijing JOINN Biologics Co. Ltd.

Electron microscopy was performed by Shuimubio Services (Beijing, China) with a Thermo Scientific TalosL120C electron microscope operated at 120 keV. SARS-CoV-2 S-TM proteins were diluted to 0.3 mg/mL in PB buffer. High-magnification images were acquired at nominal magnifications of 57,000× (0.245 nm/pixel) and 73,000× (0.192 nm/pixel), and the images were acquired at electron doses of 30e-/A2. The images were collected by a TalosL120C electron microscope, and two-dimensional classification was conducted by cisTEM software.

### BALB/c mouse immunization.

To explore the effect of dose on immunity, 136 BALB/c mice (15 to 19 g, female) were randomly grouped (*n* = 8/group). The mice were immunized intramuscularly (IM) with various doses of S-TM (1, 2.5, 5, 10, and 25 μg/dose) that were nonadjuvanted or adjuvanted with 50 μg Al (manufactured by Croda) or 50 μg Al plus 10 μg CpG (Parr Biotechnology [Jiangsu] Co., Ltd.). The total injection volume of the mixed vaccines (antigen + adjuvant) was 100 μL per IM dose. Two IM doses were administered (on days 0 and 21). The animals were bled from the cheek on day 35 for humoral immune response analyses. Serum from day 35 was neutralized against the Beta, WT, and Delta strains. The spleens were removed after euthanization on day 35 for ELISpot assays.

Another group of BALB/c mice (15 to 19 g, female) were randomly grouped (*n* = 6/group). The mice were immunized intramuscularly with various doses of S-TM (2.5, 5, and 10 μg/dose) that was nonadjuvanted or adjuvanted with 50 μg Al plus 50 μg CpG ODN (Parr Biotechnology [Jiangsu] Co., Ltd.). The immunization scheme was the same as that mentioned above. The animals were bled from the cheek on day 35 for WT, Beta, Delta, and Omicron strain SARS-CoV-2 neutralization assays.

### S-TM binding antibody ELISA.

We coated 96-well plates (Thermo, Denmark, lot number 442404) with S-TM (5 or 100 μL/well) at 4°C overnight. The plates were washed three times with PBST (phosphate-buffered saline containing 0.05% Tween 20) and then blocked with 2% bovine serum albumin (BSA) (lot number: BSA-YCSB-500g) at room temperature for 1 h. After washing the plate three times with PBST, 2-fold dilutions from 1:100 of the antisera were added to the wells (100 μL/well) and incubated at room temperature for 2 h. After the plates were washed three times with PBST, 0.09 μg/mL rabbit anti-mouse F(ab)/horseradish peroxidase (HRP) (Jackson, lot number 315-035-047) was added to the well and incubated at room temperature for 1 h. The plates were then washed three times with PBST, and signals were developed using 3,3′,5,5′-tetramethylbenzidine (TMB) substrate (Thermo Scientific) at room temperature. The colorimetric reaction was stopped after 20 min by adding 2 M HCl. The optical density (OD) was measured at 450 nm by a microplate reader (Multiskan MK3, Thermo). The 50% effective concentration (EC_50_) values were calculated by four-parameter fitting using GraphPad Prism software (GraphPad Software, Inc.).

### Live SARS-CoV-2 neutralization assay.

Heat-inactivated antisera (56°C for 30 min) from BALB/c mice and H11-K18-hACE-2 mice were serially diluted in cell culture medium in 2-fold dilutions starting from 1:25. The diluted sera (50 μL) were mixed with an equal volume of solution (50 μL) containing 100 TCID_50_ live SARS-CoV-2 VOC strain (Beta, Delta, and Omicron) and WT strain in each well. After 2 h of incubation at 37°C in a 5% CO_2_ incubator, Vero cells (1 × 10^4^/well) were added to each well and cultured in a 5% CO_2_ incubator at 37°C for 4 to 5 days. The cytopathic effect (CPE) on each well was recorded under a microscope, and the neutralization titer was calculated as the reciprocal of the maximum dilution required for 50% neutralization of viral infection by Karber’s method.

### Elispot assay and flow cytometry.

Splenocytes from immunized mice were harvested 2 weeks after the second IM immunization for multifunctional cytokine and CD4^+^/CD8^+^ T-cell analysis. To detect antigen-specific T-cell responses, ELISpot kits measuring Th1 cytokines (IFN-γ and IL-2) and Th2 cytokines (IL-4) were used per the manufacturer’s instructions (Mabtech: 3321-4AST-2, 3441-4APW-2, and 3311-4APW-2). In all, 2 × 10^5^ splenocytes were stimulated *in vitro* with 4 μg/mL SARS-CoV-2 spike peptide pool (Sinobiological, PP003). Phorbol 12-myristate 13-acetate (PMA) + 1 μg/mL ionomycin (Dakewe Biotech Co., Ltd., 2030421) and 1 μg/mL ConA (Beijing Solarbio Science & Technology Co., Ltd., C8110) as nonspecific stimuli were added to the positive-control wells, whereas the negative-control wells received no stimuli. After 20 h of incubation at 37°C, the plates were washed, and 1 μg/mL antibodies (diluted with PBS containing 0.5% FBS) were added to each well (100 μL/well). After 2 h of incubation at room temperature, the plates were washed, and 100-fold diluted streptavidin-ALP was added for 1 h of incubation at room temperature. Blots were developed by the addition of BCIP/NBT substrate solution. Deionized water was added to each well until clear blots were observed. The plates were stored in the dark, and the IFN-γ, IL-2, and IL-4 spot-forming units (SFU) were counted before the plate dried out.

For CD4 or CD8 T cells of murine splenocytes, 100 μL splenocytes were centrifuged and resuspended in 100 μL PBS. Then, 0.5 μL of 200-fold diluted PE anti-mouse CD3 (Biolegend, 100206), APC anti-mouse CD4 (Biolegend, 100412), and Alexa Fluor anti-mouse CD8 (Biolegend, 100100723) were added and mixed. After 30 min of incubation at 4°C in the dark, the mixture was centrifuged and resuspended in 200 μL PBS. All stained samples were acquired using a flow cytometer (ACEA, Novocyte 3130).

### H11-K18-hACE-2 mouse immunization and virus challenge.

Female H11-K18-hACE-2 mice (12 weeks) were randomly grouped (*n* = 9/group) and immunized by intramuscular injection with two doses spaced 21 days apart (study days 0 and 21) containing 10 μg of S-TM with 50 μg Al plus 10 μg CpG or with only PB buffer at 100 μL/dose. Serum was collected for analysis on day 34. Vaccinated mice were then transferred into the BSL-3 animal laboratory at IVDC, China CDC, on day 35 and were intranasally challenged with live SARS-CoV-2 virus Beta on day 36. For the virus challenge, vaccinated H11-K18-hACE-2 mice were anesthetized by intraperitoneal injection with 0.2 mL/10 g of tribromoethanol and then intranasally inoculated with 25 TCID_50_ of SARS-CoV-2 VOC Beta strain (GD2021) in 20 μL. The challenged mice were weighed on the day of infection and 4, 5, 6, 8, and 11 dpi. At 4, 8, and 11 dpi for the S-TM + Al + CpG-immunized group, three mice were sacrificed at each time point. For the PB buffer group, three mice were sacrificed only at 4 dpi, and the rest of the samples were collected according to the survival of the mice after 4 dpi. Lungs and brains were collected for viral load and pathology. Samples from natural death mice after infection in the PB group were collected only for viral load.

### Real-time RT-PCR for SARS-CoV-2 RNA quantification.

Tissue homogenization was performed at the BSL-3 animal laboratory at IVDC, China CDC. The tissue homogenates were clarified by centrifugation at 11,000 rpm for 5 min, and 50 μL supernatant was subjected to RNA extraction with a nucleic acid extraction kit (Xi’an Tianlong Science and Technology Co., Ltd., China). Reverse transcription-quantitative PCR (RT-qPCR) was performed using a novel coronavirus (2019-nCoV) nucleic acid detection kit (lot number: 20210810F, Shanghai BioGerm Medical Technology Co., Ltd., China) according to the protocol from the China CDC. The ORF1ab gene of SARS-CoV-2 was amplified by RT-qPCR. The gene copies indicating the viral load were quantified according to the standard (high concentration) SARS-CoV-2 nucleic acid (National Institute of Metrology, China, GBW [E] 091089).

### Histopathology analyses.

The tissues were fixed in 4% formalin and paraffin embedded. Lung sections were prepared and stained with hematoxylin and eosin (H&E). Sections were scored on a severity scale of 0 to 4 for the degree of inflammatory cell infiltration in different anatomical sites of the lung (scored 0 when no or only 1 inflammatory cell infiltration was visible, scored 1 when less than 5 infiltrations of inflammatory cells were visible, scored 2 when 5 to 10 infiltrations of inflammatory cells were visible, scored 3 when 10 to 20 infiltrations of inflammatory cells were visible, and scored 4 when more than 20 infiltrations of inflammatory cells were visible). Lung disease at different anatomical sites under light microscopy was assessed by the expansion of the parenchymal wall, interstitial pneumonia, alveolitis, and bronchiolitis.

### Statistical analysis.

Statistical analyses were performed using Prism 8.0 (GraphPad Software). Serum antibody titers were plotted for individual animals and the geometric mean titer (GMT) and 95% confidence interval (95% CI) or the means ± standard error of the mean (SEM) as indicated in the figure. RNA copies, lung pathology scores, and splenocytes of different cytokines are presented as mean SEM. Comparisons were performed using ordinary one-way or two-way analysis of variance (ANOVA) with Tukey’s multiple-comparison test. *P* values < 0.05 were considered significant. “ns” indicates no significance.

## References

[1] Choi J, Smith D. 2021. SARS-CoV-2 variants of concern. Yonsei Med J 62:961–968. doi:10.3349/ymj.2021.62.11.961. 34672129 PMC8542474

[2] Bian L, Gao Q, Gao F, Wang Q, He Q, Wu X, Mao Q, Xu M, Liang Z. 2021. Impact of the Delta variant on vaccine efficacy and response strategies. Expert Rev Vaccines 20:1201–1209. doi:10.1080/14760584.2021.1976153. 34488546 PMC8442750

[3] Carreño JM, Alshammary H, Tcheou J, Singh G, Raskin AJ, Kawabata H, Sominsky LA, Clark JJ, Adelsberg DC, Bielak DA, Gonzalez-Reiche AS, Dambrauskas N, Vigdorovich V, Alburquerque B, Amoako AA, Banu R, Beach KF, Bermúdez-González MC, Cai GY, Ceglia I, Cognigni C, Farrugia K, Gleason CR, van de Guchte A, Kleiner G, Khalil Z, Lyttle N, Mendez WA, Mulder LCF, Oostenink A, Rooker A, Salimbangon AT, Saksena M, Paniz-Mondolfi AE, Polanco J, Srivastava K, Sather DN, Sordillo EM, Bajic G, van Bakel H, Simon V, Krammer F, PSP-PARIS Study Group. 2022. Activity of convalescent and vaccine serum against SARS-CoV-2 Omicron. Nature 602:682–688. doi:10.1038/d41586-021-03846-z. 35016197

[4] Chen Z, Zhang Y, Wang M, Islam MS, Liao P, Hu Y, Chen X. 2022. Humoral and cellular immune responses of COVID-19 vaccines against SARS-Cov-2 Omicron variant: a systemic review. Int J Biol Sci 18:4629–4641. doi:10.7150/ijbs.73583. 35874952 PMC9305266

[5] Gupta SL, Mantus G, Manning KE, Ellis M, Patel M, Ciric CR, Lu A, Turner JS, O’Halloran JA, Presti RM, Joshi DJ, Ellebedy AH, Anderson EJ, Rostad CA, Suthar MS, Wrammert J. 2022. Loss of Pfizer (BNT162b2) vaccine-induced antibody responses against the SARS-CoV-2 Omicron variant in adolescents and adults. J Virol 96:e0058222. doi:10.1128/jvi.00582-22. 35976000 PMC9472620

[6] Melo-González F, Soto J, González L, Fernández J, Duarte L, Schultz B, Gálvez N, Pacheco G, Ríos M, Vázquez Y, Rivera-Pérez D, Moreno-Tapia D, Iturriaga C, Vallejos O, Berríos-Rojas R, Hoppe-Elsholz G, Urzúa M, Bruneau N, Fasce R, Mora J, Grifoni A, Sette A, Weiskopf D, Zeng G, Meng W, González-Aramundiz J, González P, Abarca K, Ramírez E, Kalergis A, Bueno S. 2021. Recognition of variants of concern by antibodies and T cells induced by a SARS-CoV-2 inactivated vaccine. Front Immunol 12:747830. doi:10.3389/fimmu.2021.747830. 34858404 PMC8630786

[7] Woldemeskel B, Garliss C, Blankson J. 2021. SARS-CoV-2 mRNA vaccines induce broad CD4+ T cell responses that recognize SARS-CoV-2 variants and HCoV-NL63. J Clin Invest 131:e149335. doi:10.1172/JCI149335. 33822770 PMC8121504

[8] Sette A, Crotty S. 2021. Adaptive immunity to SARS-CoV-2 and COVID-19. Cell 184:861–880. doi:10.1016/j.cell.2021.01.007. 33497610 PMC7803150

[9] Lu F, Mosley YC, Carmichael B, Brown DD, HogenEsch H. 2010. Formulation of aluminum hydroxide adjuvant with TLR agonists poly(I:C) and CpG enhances the magnitude and avidity of the humoral immune response. Vaccine 37:1945–1953. 10.1016/j.vaccine.2019.02.033 30803844

[10] Nanishi E, Borriello F, O’Meara T, McGrath M, Saito Y, Haupt R, Seo H, van Haren S, Cavazzoni C, Brook B, Barman S, Chen J, Diray-Arce J, Doss-Gollin S, De Leon M, Prevost-Reilly A, Chew K, Menon M, Song K, Xu A, Caradonna T, Feldman J, Hauser B, Schmidt A, Sherman A, Baden L, Ernst R, Dillen C, Weston S, Johnson R, Hammond H, Mayer R, Burke A, Bottazzi M, Hotez P, Strych U, Chang A, Yu J, Sage P, Barouch D, Dhe-Paganon S, Zanoni I, Ozonoff A, Frieman M, Levy O, Dowling D. 2022. An aluminum hydroxide:CpG adjuvant enhances protection elicited by a SARS-CoV-2 receptor binding domain vaccine in aged mice. Sci Transl Med 14:eabj5305. doi:10.1126/scitranslmed.abj5305. 34783582 PMC10176044

[11] Meier S, Guthe S, Kiefhaber T, Grzesiek S. 2004. Foldon, the natural trimerization domain of T4 fibritin, dissociates into a monomeric A-state form containing a stable beta-hairpin: atomic details of trimer dissociation and local beta-hairpin stability from residual dipolar couplings. J Mol Biol 344:1051–1069. doi:10.1016/j.jmb.2004.09.079. 15544812

[12] Wrapp D, Wang N, Corbett K, Goldsmith J, Hsieh C, Abiona O, Graham B, McLellan J. 2020. Cryo-EM structure of the 2019-nCoV spike in the prefusion conformation. bioRxiv. doi:10.1101/2020.02.11.944462. PMC7164637 32075877

[13] Ma J, Su D, Sun Y, Huang X, Liang Y, Fang L, Ma Y, Li W, Liang P, Zheng S. 2021. Cryo-EM structure of S-Trimer, a subunit vaccine candidate for COVID-19. J Virol 95:e00194-21. doi:10.1128/JVI.00194-21. 33692215 PMC8139713

[14] Tada R, Muto S, Iwata T, Hidaka A, Kiyono H, Kunisawa J, Aramaki Y. 2017. Attachment of class B CpG ODN onto DOTAP/DC-chol liposome in nasal vaccine formulations augments antigen-specific immune responses in mice. BMC Res Notes 10:68. doi:10.1186/s13104-017-2380-8. 28126014 PMC5270218

[15] Song S, Zhou B, Cheng L, Liu W, Fan Q, Ge X, Peng H, Fu Y, Ju B, Zhang Z. 2022. Sequential immunization with SARS-CoV-2 RBD vaccine induces potent and broad neutralization against variants in mice. Virol J 19:2. doi:10.1186/s12985-021-01737-3. 34983583 PMC8724645

[16] Pavot V, Berry C, Kishko M, Anosova N, Huang D, Tibbitts T, Raillard A, Gautheron S, Gutzeit C, Koutsoukos M, Chicz R, Lecouturier V. 2022. Protein-based SARS-CoV-2 spike vaccine booster increases cross-neutralization against SARS-CoV-2 variants of concern in non-human primates. Nat Commun 13:1699. doi:10.1038/s41467-022-29219-2. 35361754 PMC8971430

[17] Wu K, Choi A, Koch M, Elbashir S, Ma L, Lee D, Woods A, Henry C, Palandjian C, Hill A, Jani H, Quinones J, Nunna N, O’Connell S, McDermott A, Falcone S, Narayanan E, Colpitts T, Bennett H, Corbett K, Seder R, Graham B, Stewart-Jones G, Carfi A, Edwards D. 2021. Variant SARS-CoV-2 mRNA vaccines confer broad neutralization as primary or booster series in mice. Vaccine 39:7394–7400. doi:10.1016/j.vaccine.2021.11.001. 34815117 PMC8572694

[18] Fischer RJ, van Doremalen N, Adney DR, Yinda CK, Port JR, Holbrook MG, Schulz JE, Williamson BN, Thomas T, Barbian KD, Anzick SL, Ricklefs SM, Smith BJ, Long D, Martens C, Saturday G, de Wit E, Gilbert SC, Lambe T, Munster VJ. 2021. ChAdOx1 nCoV-19 (AZD1222) protects Syrian hamsters against SARS-CoV-2 B.1.351 and B.1.1.7. Nat Commun 12:5868. doi:10.1038/s41467-021-26178-y. 34620866 PMC8497486

[19] Debes AK, Xiao S, Egbert ER, Caturegli P, Sitaras I, Pekosz A, Milstone AM. 2022. Comparison of total and neutralizing SARS-CoV-2 spike antibodies against Omicron and other variants in paired samples after two or three doses of mRNA vaccine. medRxiv. doi:10.1101/2022.01.26.22269819. PMC9603442 36190405

[20] Gagne M, Moliva JI, Foulds KE, Andrew SF, Flynn BJ, Werner AP, Wagner DA, Teng I-T, Lin BC, Moore C, Jean-Baptiste N, Carroll R, Foster SL, Patel M, Ellis M, Edara V-V, Maldonado NV, Minai M, McCormick L, Honeycutt CC, Nagata BM, Bock KW, Dulan CNM, Cordon J, Todd J-PM, McCarthy E, Pessaint L, Van Ry A, Narvaez B, Valentin D, Cook A, Dodson A, Steingrebe K, Flebbe DR, Nurmukhambetova ST, Godbole S, Henry AR, Laboune F, Roberts-Torres J, Lorang CG, Amin S, Trost J, Naisan M, Basappa M, Willis J, Wang L, Shi W, Doria-Rose NA, Olia AS, Liu C. 2022. mRNA-1273 or mRNA-Omicron boost in vaccinated macaques elicits comparable B cell expansion, neutralizing antibodies and protection against Omicron. bioRxiv. doi:10.1101/2022.02.03.479037. PMC8947944 35447072

[21] Qu L, Yi Z, Shen Y, Lin L, Chen F, Xu Y, Wu Z, Tang H, Zhang X, Tian F, Wang C, Xiao X, Dong X, Guo L, Lu S, Yang C, Tang C, Yang Y, Yu W, Wang J, Zhou Y, Huang Q, Yisimayi A, Cao Y, Wang Y, Zhou Z, Peng X, Wang J, Xie XS, Wei W. 2022. Circular RNA vaccines against SARS-CoV-2 and emerging variants. bioRxiv. doi:10.1101/2021.03.16.435594. PMC8971115 35460644

[22] Tubiana J, Xiang Y, Fan L, Wolfson HJ, Chen K, Schneidman-Duhovny D, Shi Y. 2022. Reduced antigenicity of Omicron lowers host serologic response. Cell Rep 41:111512. doi:10.1016/j.celrep.2022.111512. 36223774 PMC9515332

[23] Dejnirattisai W, Huo J, Zhou D, Zahradník J, Supasa P, Liu C, Duyvesteyn HM, Ginn HM, Mentzer AJ, Tuekprakhon A, Nutalai R, Wang B, Dijokaite A, Khan S, Avinoam O, Bahar M, Skelly D, Adele S, Johnson SA, Amini A, Ritter TG, Mason C, Dold C, Pan D, Assadi S, Bellass A, Omo-Dare N, Koeckerling D, Flaxman A, Jenkin D, Aley PK, Voysey M, Costa Clemens SA, Naveca FG, Nascimento V, Nascimento F, Fernandes da Costa C, Resende PC, Pauvolid-Correa A, Siqueira MM, Baillie V, Serafin N, Kwatra G, Da Silva K, Madhi SA, Nunes MC, Malik T, Openshaw PJ, Baillie JK, Semple MG, et al. 2022. SARS-CoV-2 Omicron-B.1.1.529 leads to widespread escape from neutralizing antibody responses. Cell 185:467–484.e15. doi:10.1016/j.cell.2021.12.046. 35081335 PMC8723827

[24] Li C, Zhan W, Yang Z, Tu C, Hu G, Zhang X, Song W, Du S, Zhu Y, Huang K, Kong Y, Zhang M, Mao Q, Gu X, Zhang Y, Xie Y, Deng Q, Song Y, Chen Z, Lu L, Jiang S, Wu Y, Sun L, Ying T. 2022. Broad neutralization of SARS-CoV-2 variants by an inhalable bispecific single-domain antibody. Cell 185:1389–1401.e18. doi:10.1016/j.cell.2022.03.009. 35344711 PMC8907017

[25] Logue J, Johnson R, Patel N, Zhou B, Maciejewski S, Zhou H, Portnoff A, Tian J-H, McGrath M, Haupt R, Weston S, Hammond H, Guebre-Xabier M, Dillen C, Plested J, Cloney-Clark S, Greene AM, Massare M, Glenn G, Smith G, Frieman M. 2021. Immunogenicity and *in vivo* protection of a variant nanoparticle vaccine that confers broad protection against emerging SARS-CoV-2 variants. bioRxiv. doi:10.1101/2021.06.08.447631. PMC9972327 36854666

[26] Su D, Li X, He C, Huang X, Chen M, Wang Q, Qin W, Liang Y, Xu R, Wu J, Luo P, Yang X, Zeng Y, Luo M, Luo D, Salisbury DM, Ambrosino D, Siber G, Clemens R, Liang P, Liang JG. 2021. Broad neutralization against SARS-CoV-2 variants induced by a modified B.1.351 protein-based COVID-19 vaccine candidate. bioRxiv. doi:10.1101/2021.05.16.444369.

[27] Kim H, Canchola J, Brandt C, Pyles G, Chanock R, Jensen K, Parrott R. 1969. Respiratory syncytial virus disease in infants despite prior administration of antigenic inactivated vaccine. Am J Epidemiol 89:422–434. doi:10.1093/oxfordjournals.aje.a120955. 4305198

[28] Fulginiti V, Eller J, Downie A, Kempe C. 1967. Altered reactivity to measles virus. Atypical measles in children previously immunized with inactivated measles virus vaccines. JAMA 202:1075–1080. doi:10.1001/jama.202.12.1075. 6072745

[29] Tan A, Linster M, Tan C, Le Bert N, Chia W, Kunasegaran K, Zhuang Y, Tham C, Chia A, Smith G, Young B, Kalimuddin S, Low J, Lye D, Wang L, Bertoletti A. 2021. Early induction of functional SARS-CoV-2-specific T cells associates with rapid viral clearance and mild disease in COVID-19 patients. Cell Rep 34:108728. doi:10.1016/j.celrep.2021.108728. 33516277 PMC7826084

[30] Rydyznski Moderbacher C, Ramirez S, Dan J, Grifoni A, Hastie K, Weiskopf D, Belanger S, Abbott R, Kim C, Choi J, Kato Y, Crotty E, Kim C, Rawlings S, Mateus J, Tse L, Frazier A, Baric R, Peters B, Greenbaum J, Ollmann Saphire E, Smith D, Sette A, Crotty S. 2020. Antigen-specific adaptive immunity to SARS-CoV-2 in acute COVID-19 and associations with age and disease severity. Cell 183:996–1012.e19. doi:10.1016/j.cell.2020.09.038. 33010815 PMC7494270

[31] Sekine T, Perez-Potti A, Rivera-Ballesteros O, Strålin K, Gorin J, Olsson A, Llewellyn-Lacey S, Kamal H, Bogdanovic G, Muschiol S, Wullimann D, Kammann T, Emgård J, Parrot T, Folkesson E, Rooyackers O, Eriksson L, Henter J, Sönnerborg A, Allander T, Albert J, Nielsen M, Klingström J, Gredmark-Russ S, Björkström N, Sandberg J, Price D, Ljunggren H, Aleman S, Buggert M, Karolinska COVID-19 Study Group. 2020. Robust T cell immunity in convalescent individuals with asymptomatic or mild COVID-19. Cell 183:158–168.e14. doi:10.1016/j.cell.2020.08.017. 32979941 PMC7427556

[32] Grifoni A, Weiskopf D, Ramirez S, Mateus J, Dan J, Moderbacher C, Rawlings S, Sutherland A, Premkumar L, Jadi R, Marrama D, de Silva A, Frazier A, Carlin A, Greenbaum J, Peters B, Krammer F, Smith D, Crotty S, Sette A. 2020. Targets of T cell responses to SARS-CoV-2 coronavirus in humans with COVID-19 disease and unexposed individuals. Cell 181:1489–1501.e15. doi:10.1016/j.cell.2020.05.015. 32473127 PMC7237901

[33] Ahmed S, Quadeer A, McKay M. 2022. SARS-CoV-2 T cell responses elicited by COVID-19 vaccines or infection are expected to remain robust against Omicron. Viruses 14:79. doi:10.3390/v14010079. 35062283 PMC8781795

[34] Keeton R, Tincho M, Ngomti A, Baguma R, Benede N, Suzuki A, Khan K, Cele S, Bernstein M, Karim F, Madzorera S, Moyo-Gwete T, Mennen M, Skelem S, Adriaanse M, Mutithu D, Aremu O, Stek C, Du Bruyn E, Van Der Mescht M, de Beer Z, de Villiers T, Bodenstein A, van den Berg G, Mendes A, Strydom A, Venter M, Giandhari J, Naidoo Y, Pillay S, Tegally H, Grifoni A, Weiskopf D, Sette A, Wilkinson R, de Oliveira T, Bekker L, Gray G, Ueckermann V, Rossouw T, Boswell M, Bihman J, Moore P, Sigal A, Ntusi N, Burgers W, Riou C. 2022. T cell responses to SARS-CoV-2 spike cross-recognize Omicron. Nature 603:488–492. doi:10.1038/s41586-022-04460-3. 35102311 PMC8930768

[35] Liu J, Chandrashekar A, Sellers D, Barrett J, Lifton M, McMahan K, Sciacca M, VanWyk H, Wu C, Yu J, Collier A, Barouch D. 2022. Vaccines elicit highly cross-reactive cellular immunity to the SARS-CoV-2 Omicron variant. medRxiv. doi:10.1101/2022.01.02.22268634. PMC8930761 35102312

[36] Tarke A, Coelho C, Zhang Z, Dan J, Yu E, Methot N, Bloom N, Goodwin B, Phillips E, Mallal S, Sidney J, Filaci G, Weiskopf D, da Silva Antunes R, Crotty S, Grifoni A, Sette A. 2022. SARS-CoV-2 vaccination induces immunological T cell memory able to cross-recognize variants from Alpha to Omicron. Cell 185:847–859.e11. doi:10.1016/j.cell.2022.01.015. 35139340 PMC8784649

[37] GeurtsvanKessel CA-OX, Geers DA-O, Schmitz KA-O, Mykytyn AA-O, Lamers MA-O, Bogers SA-O, Scherbeijn S, Gommers LA-O, Sablerolles RA-O, Nieuwkoop NN, Rijsbergen LA-O, van Dijk LA-O, de Wilde J, Alblas KA-O, Breugem TI, Rijnders BA-O, de Jager HA-O, Weiskopf DA-O, van der Kuy PA-O, Sette AA-O, Koopmans MA-O, Grifoni AA-O, Haagmans BA-O, de Vries RA-O. 2022. Divergent SARS-CoV-2 Omicron-reactive T and B cell responses in COVID-19 vaccine recipients. Sci Immunol 7:eabo2202. doi:10.1126/sciimmunol.abo2202. 35113647 PMC8939771

[38] Yinda C, Port J, Bushmaker T, Offei Owusu I, Purushotham J, Avanzato V, Fischer R, Schulz J, Holbrook M, Hebner M, Rosenke R, Thomas T, Marzi A, Best S, de Wit E, Shaia C, van Doremalen N, Munster V. 2021. K18-hACE2 mice develop respiratory disease resembling severe COVID-19. PLoS Pathog 17:e1009195. doi:10.1371/journal.ppat.1009195. 33465158 PMC7875348

[39] Kumari P, Rothan H, Natekar J, Stone S, Pathak H, Strate P, Arora K, Brinton M, Kumar M. 2021. Neuroinvasion and encephalitis following intranasal inoculation of SARS-CoV-2 in K18-hACE2 Mice. Viruses 13:132. doi:10.3390/v13010132. 33477869 PMC7832889

[40] Radvak P, Kwon H, Kosikova M, Ortega-Rodriguez U, Xiang R, Phue J, Shen R, Rozzelle J, Kapoor N, Rabara T, Fairman J, Xie H. 2021. SARS-CoV-2 B.1.1.7 (alpha) and B.1.351 (beta) variants induce pathogenic patterns in K18-hACE2 transgenic mice distinct from early strains. Nat Commun 12:6559. doi:10.1038/s41467-021-26803-w. 34772941 PMC8589842

[41] Yang A, Kern F, Losada P, Agam M, Maat C, Schmartz G, Fehlmann T, Stein J, Schaum N, Lee D, Calcuttawala K, Vest R, Berdnik D, Lu N, Hahn O, Gate D, McNerney M, Channappa D, Cobos I, Ludwig N, Schulz-Schaeffer W, Keller A, Wyss-Coray T. 2021. Dysregulation of brain and choroid plexus cell types in severe COVID-19. Nature 595:565–571. doi:10.1038/s41586-021-03710-0. 34153974 PMC8400927

[42] Mathieu E, Ritchie H, Ortiz-Ospina E, Roser M, Hasell J, Appel C, Giattino C, Rodes-Guirao L. 2021. A global database of COVID-19 vaccinations. Nat Hum Behav 5:947–953. doi:10.1038/s41562-021-01122-8. 33972767

[43] World Health Organization. 2021. Achieving 70% COVID-19 immunization coverage by Mid-2022. https://www.who.int/news/item/23-12-2021-achieving-70-covid-19-immunization-coverage-by-mid-2022.

[44] Choi A, Koch M, Wu K, Chu L, Ma L, Hill A, Nunna N, Huang W, Oestreicher J, Colpitts T, Bennett H, Legault H, Paila Y, Nestorova B, Ding B, Montefiori D, Pajon R, Miller JM, Leav B, Carfi A, McPhee R, Edwards DK. 2021. Safety and immunogenicity of SARS-CoV-2 variant mRNA vaccine boosters in healthy adults: an interim analysis. Nat Med 27:2025–2031. doi:10.1038/s41591-021-01527-y. 34526698 PMC8604720

[45] Launay O, Cachanado M, Luong Nguyen LB, Ninove L, Lachatre M, Ben Ghezala I, Bardou M, Schmidt-Mutter C, Lacombe K, Laine F, Allain JS, Botelho-Nevers E, Tavolacci MP, Chidiac C, Pavese P, Dussol B, Priet S, Deplanque D, Touati A, Curci L, Konate E, Ben Hamouda N, Besbes A, Nubret E, Capelle F, Berard L, Rousseau A, Tartour E, Simon T, de Lamballerie X. 2022. Immunogenicity and safety of beta-adjuvanted recombinant booster vaccine. N Engl J Med 387:374–376. doi:10.1056/NEJMc2206711. 35767474 PMC9258749

