# Supplementary material for: A Beta Strain-Based Spike Glycoprotein Vaccine Candidate Induces Broad Neutralization and Protection against SARS-CoV-2 Variants of Concern
Source: Microbiol Spectr. 2023 Feb 27;11(2):e02687-22. doi: 10.1128/spectrum.02687-22 (PMC10100794; doi:10.1128/spectrum.02687-22)
Supplement: Supplemental file 1 — Supplemental material. Download spectrum.02687-22-s0001.pdf, PDF file, 1.7 MB [file spectrum.02687-22-s0001.pdf]

A Beta strain-based spike glycoprotein vaccine candidate induces broad neutralization and protection against SARS-CoV-2 variants of concern

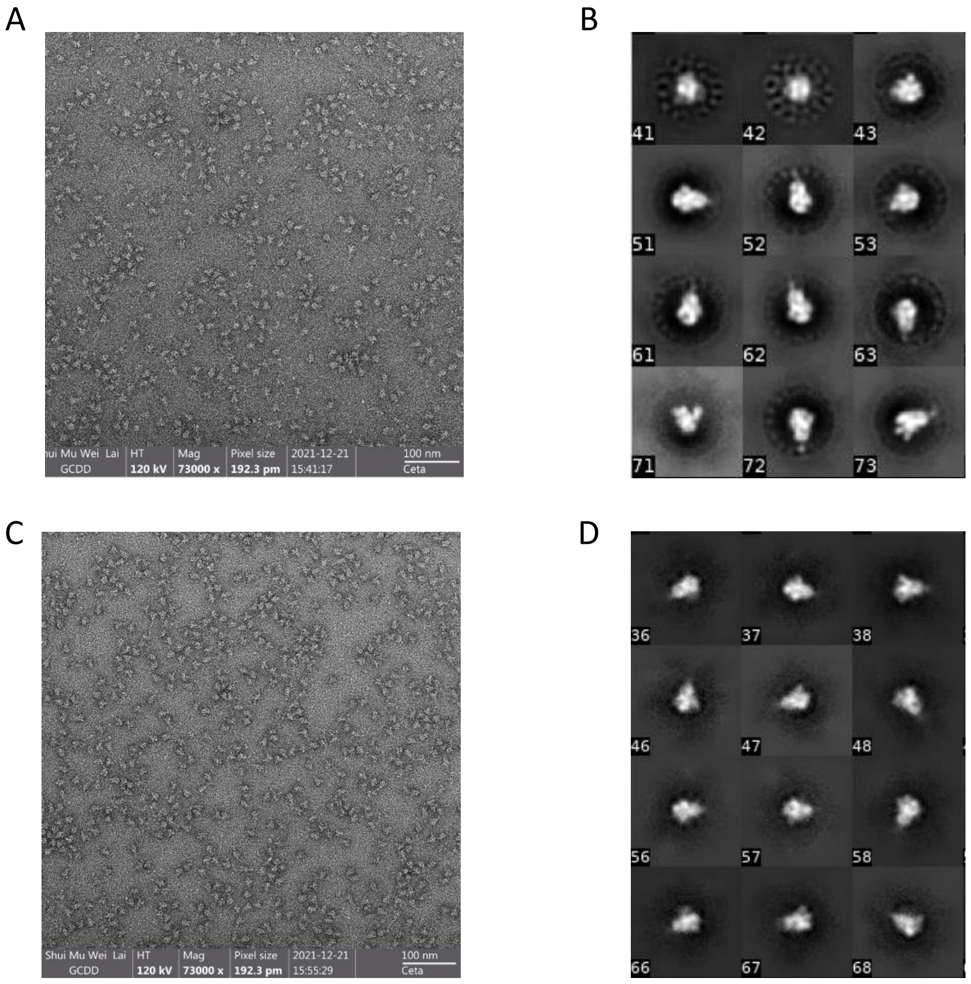

Fig S1. Structural of the S-TM showed a homogeneous trimeric structure with good dispersion when stored in different condition. Negative-stain EM images (A) and 2D images (B) of S-TM when stored in 4℃ for 87 days. Negative-stain EM images (C) and 2D images (D) of S-TM when stored in -80 ℃ for 112 days.

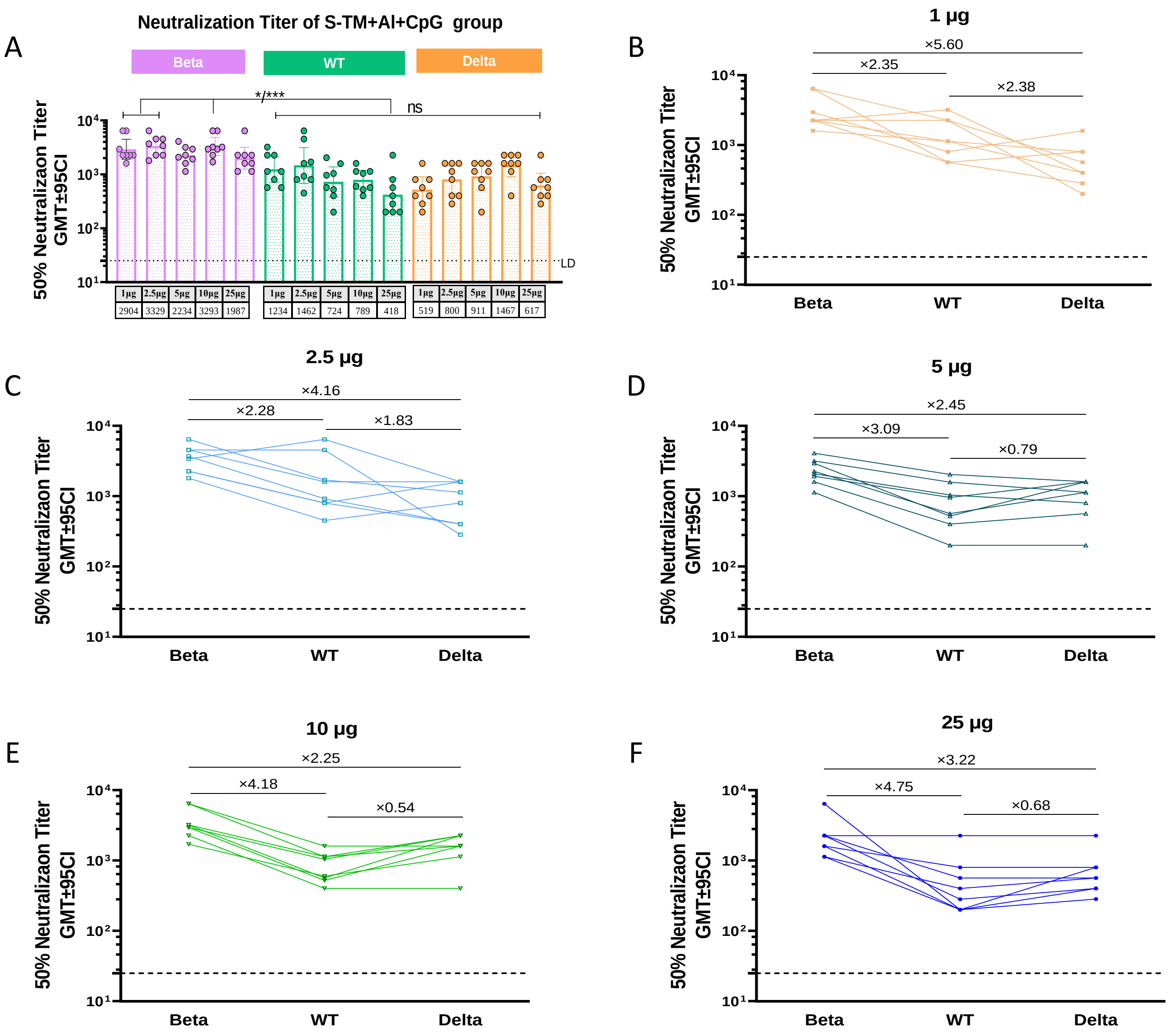

Fig S2. Comparison of neutralization antibody titers with live SARS-CoV-2 assay against different strains in S-TM+Al+CpG group(n=8). Neutralizing antibodies against beta, WT and Delta were detected 14 days after vaccination (A) with 1μg (B) , 2.5μg (C), 5μg (D), 10μg (E) and 25μg(F) S-TM that with 50ug Al+10ug CpG. The limitation of detection (LD) was initial dilution fold (1:25), neutralization titers lower than LD were considered as initial dilution and were used as fold limited detection for calculation and plotting. Points represent individual mouse.

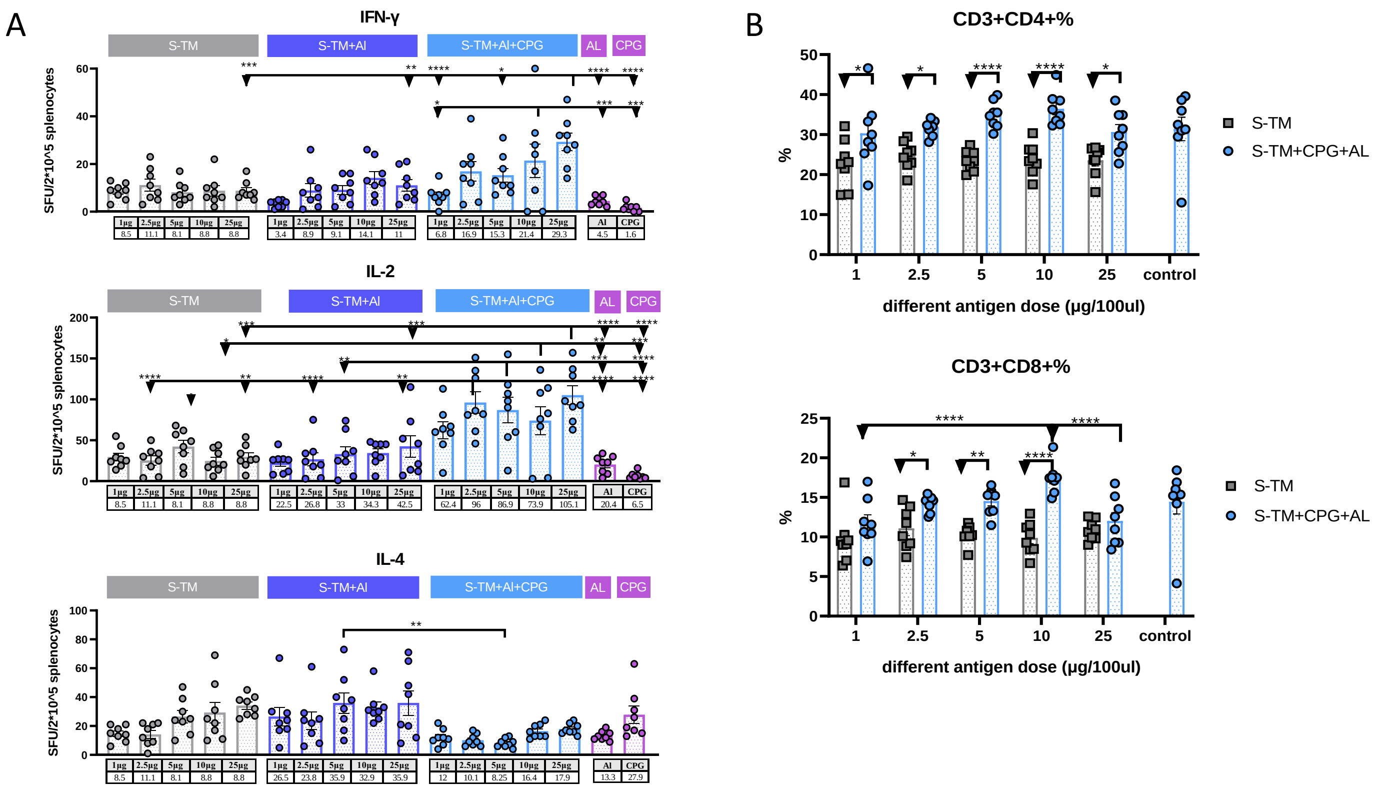

Fig S3. Cell-mediated immunity upon immunization with S-TM based on Beta variant. BALB/c mice (n=8) were immunized with 1,2.5,5,10 and 25μg S-TM that was non-adjuvanted or adjuvanted with 50ug Al or 50ug Al +10ug CpG twice on Day 0 and Day 21, different dose group was shown. A. The amount of immune cells in spleen secreting IL-2, IFN-γ, IL-4 cytokines by ELISpot was determined 14 days post second vaccination. B. The frequency of CD3+CD4+ and CD3+CD8+ T cells in different group. Bars indicate Mean±SEM. Comparisons were made using one way ANOVA with Tukey's multiple comparisons test. P values < 0.05 were considered significant.

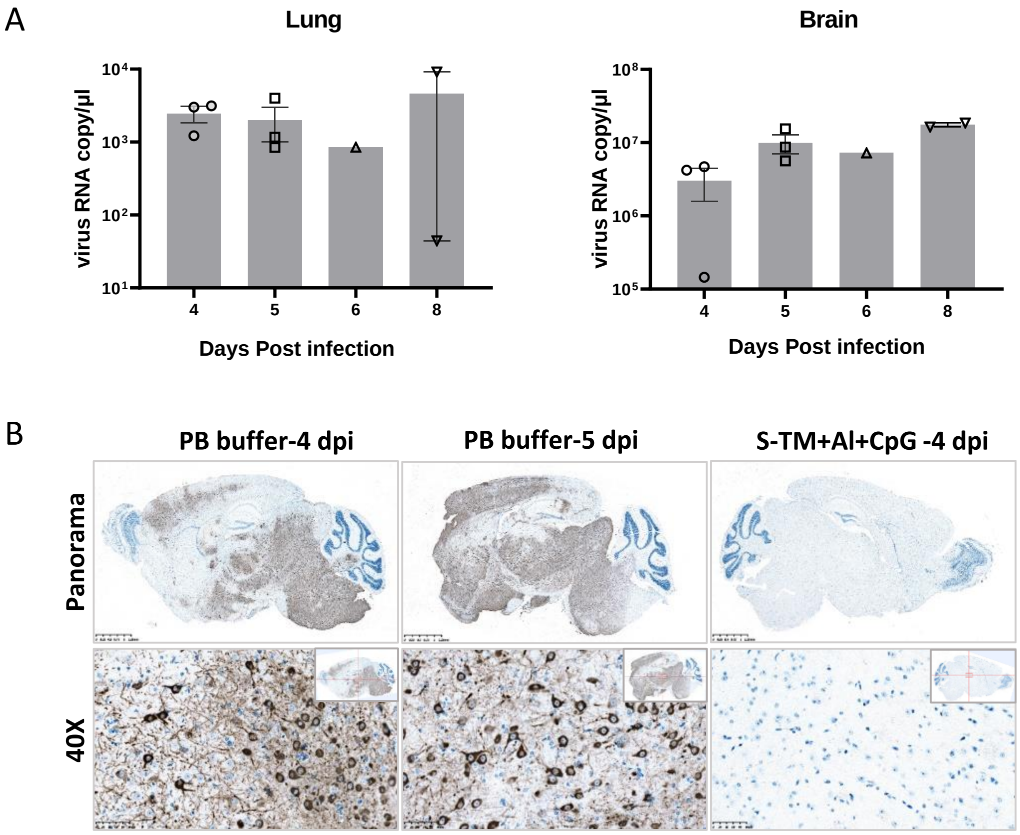

Fig S4. Viral loads based on RNA copies of Lung and brain in Control group (PB group), IHC analysis for SARS-CoV-2 NP antigen in the brain of H11-K18-hACE-2 mice(n=9) post infection after immunization with S-TM based on Beta variant. A. Lung and brain tissues were sampled at 4, 5, 6 and 8 days for RT-PCR detection according to the natural death of unvaccinated mice infected with Beta strain. B. IHC staining with NP antibody to SARS-CoV-2 Spike protein was conducted. IHC images shows distribution of viral NP mainly in Cerebrum and thalamus at 4 dpi and significantly spread throughout the whole brain besides cerebellum at 5 dpi in Control group. However, no distribution of viral NP in the brain was observed in S-TM+Al+CpG group at 4 dpi.
